# Oncolytic adenovirus inhibits TNBC tumor growth/metastasis in mice by targeting TGF-β and overexpressing GM-CSF

**DOI:** 10.1016/j.omton.2025.201015

**Published:** 2025-07-28

**Authors:** Nguyễn Thị Thanh Nhàn, Soon Cheon Shin, Beniamin Filimon, Yuefeng Yang, Zebin Hu, Bruce Brockstein, Weidong Xu

## Main text

(Molecular Therapy: Oncology *33*, 200936; March 2025)

During proof stage, an error during revision of the article title resulted in the term “TGF-β” being erroneously written as “TGFB.” The title has now been updated online. The editors apologize for this error.

