# Supplementary material for: Oncolytic adenovirus inhibits TNBC tumor growth/metastasis in mice by targeting TGF-β and overexpressing GM-CSF
Source: Mol Ther Oncol. 2025 Jan 17;33(1):200936. doi: 10.1016/j.omton.2025.200936 (PMC11919423; doi:10.1016/j.omton.2025.200936)
Supplement: Document S1. Figures S1–S3 and Tables S1 and S2 [file mmc1.pdf]

OMTON, Volume *33*

Supplemental information

Oncolytic adenovirus inhibits TNBC tumor growth/metastasis in mice by targeting TGF-β and overexpressing GM-CSF

Nguyễn Thị Thanh Nhàn, Soon Cheon Shin, Beniamin Filimon, Yuefeng Yang, Zebin Hu, Bruce Brockstein, and Weidong Xu

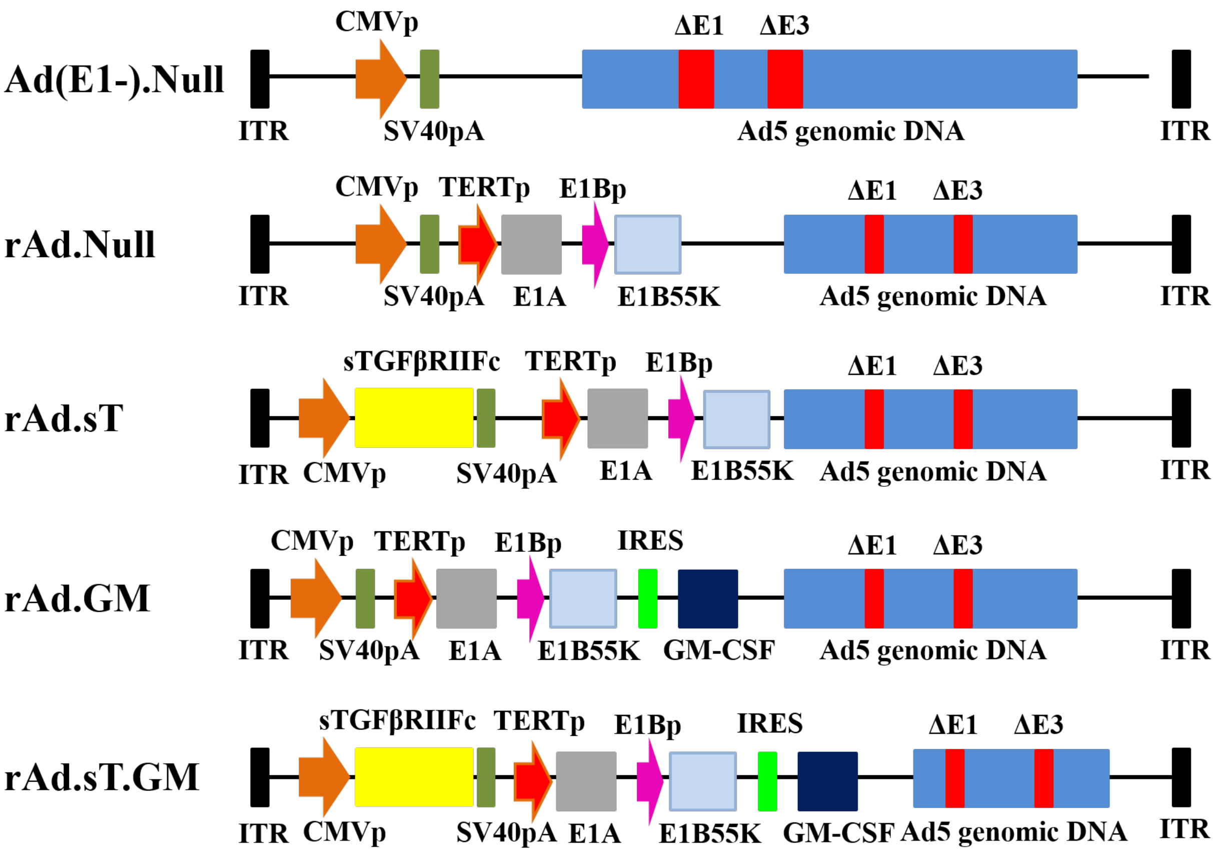

Figure S1: A schematic diagram of key genes in Ad(E-).null, rAd.null, rAd.sT, rAd.GM, and rAd.sT.GM

ITR: Inverted Terminal Repeat containing the cis-acting DNA sequences that is important in the initiation of viral DNA replication and binding to the cellular transcription factors SP1 and ATF;

CMVp: human cytomegalovirus (CMV) promoter for target gene expression;

SV40 pA: a SV40 polyadenylation site increasing mRNA stability;

TERTp: Telomerase reverse transcriptase (TERT) promoter allowing target gene expression in cells with high telomerase activity;

E1A: Adenovirus early region 1A (E1A) producing E1A protein to induce DNA replication;

E1Bp: Adenovirus early region 1B (E1B) promoter allowing E1B55K expression;

E1B55K: The gene for 55-kDa E1B protein that supports productive adenoviral infection and

replication;

IRES: Internal ribosome entry site allowing the expression of additional target genes. It is an RNA element that allows for translation initiation in the absence of a 5' cap for stable expression in cells by viral vectors;

ΔE1: The deletion of the early region 1 (E1) gene which encodes E1A and E1B proteins required for adenoviral replication and a productive lytic cycle.

ΔE3: The deletion of the early region 3 (E3) gene that expresses E3 proteins that manipulate the host immune response for immune evasion.

The expression of the therapeutic (target) genes: sTGFβRIIFc and GM-CSF is mediated either by the CMV promoter or IRES, respectively.

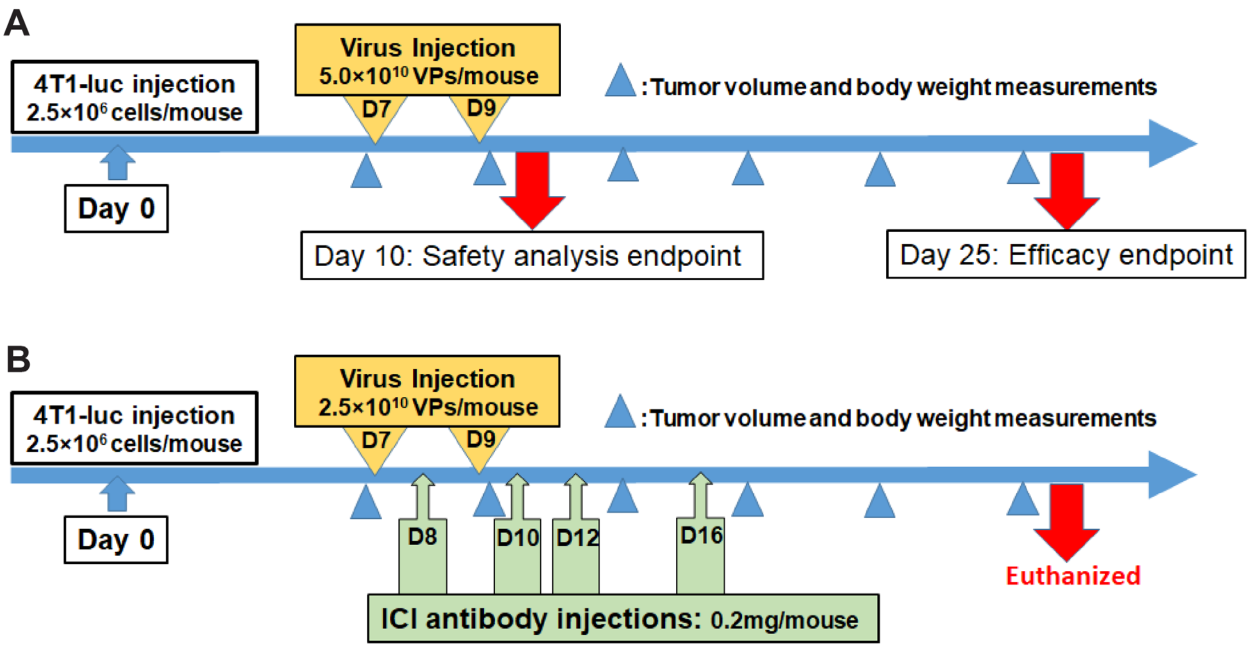

Figure S2: Treatment regimens. (A) The single agent study: Female BALB/c mice (6-8 weeks old) were subcutaneously injected with 2.0×10^6 4T1-luc cells per mouse into the dorsal right flank on day 0. Tumors became visible by day 6. On days 7 and 9 post-tumor cell inoculation, two doses of saline or adenoviruses (5.0×10^{10} VPs per mouse each injection) were injected directly into the tumors. 5 mice per treatment group were sacrificed on day 10 for safety analysis, and 12-13 mice per group were monitored with animal health conditions and periodic tumor caliper for tumor sizes, and sacrificed on Day 25 for the efficacy study. At all terminal points, blood, tumor and tissue samples were collected for analysis; (B) The combination study: Using the same animal model with a reduced dose of rAd.sT.GM (2.5×10^{10} VPs per mouse each injection), and with the combination of anti-PD-1 antibody, anti-CTLA-4 antibodies, or both (0.2 mg of antibody per mouse each injection, IP, on days 8, 10, 12, and 16), the efficacy of the combination treatment was evaluated. 11-12 mice per treatment group and all of them were carefully monitored for their health conditions and tumor volumes. They were sacrificed on Day 25 with specimens collected as described above.

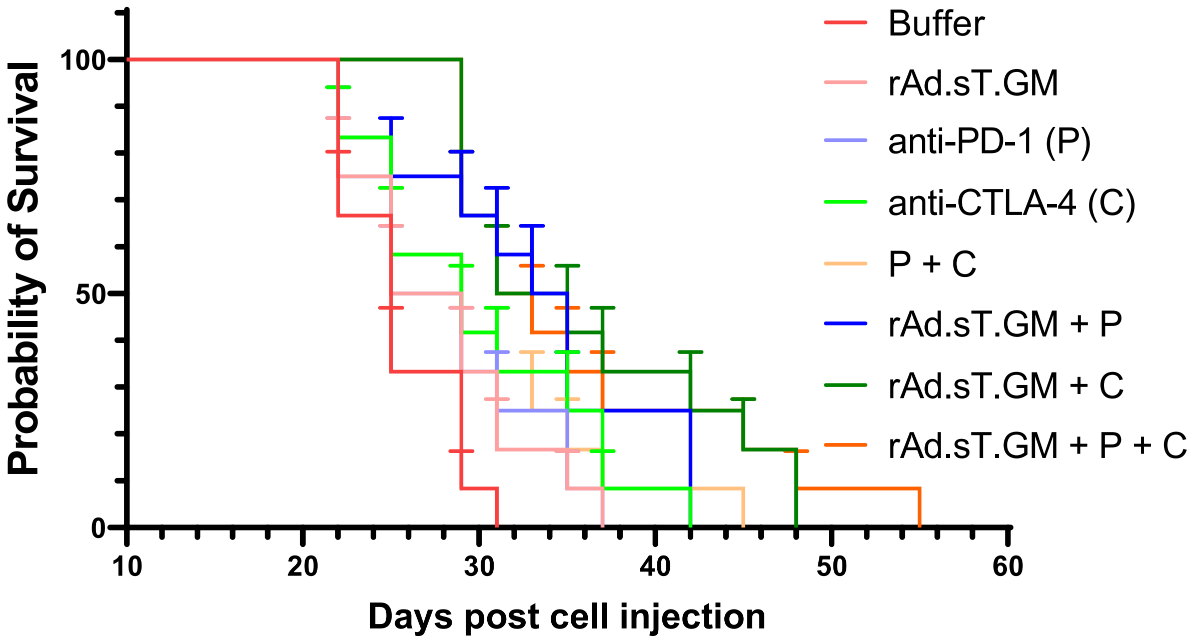

Figure S3: Kaplan-Meier curves displaying the overall survival of subjects across different treatment groups (n=12). Survival data were statistically analyzed using the Log-rank (Mantel-Cox) tests, with significant differences between the Buffer control and treatment groups indicated below:

Buffer vs anti-CTLA-4: $p = 0.0369$ (*)

Buffer vs anti-PD-1 + anti-CTLA-4: $p = 0.0369$ (*)

Buffer vs rAd.sT.GM + anti-PD-1: $p = 0.0019$ (**)

Buffer vs rAd.sT.GM + anti-CTLA-4: $p = 0.0001$ (***)

Buffer vs rAd.sT.GM + anti-PD-1 + anti-CTLA-4: $p = 0.0001$ (***)

Significance is indicated as *= $p < 0.05$, **= $p < 0.01$, ***= $p < 0.001$.

Table S1: Comparsions of treatment efficacy of rAd.sT.GM to other treatments and the buffer group in the single agent study. Significance is indicated as *= $p < 0.05$, **= $p < 0.01$, ***= $p < 0.001$, ****= $p < 0.0001$.

Measurements	Statistical analysis methods	rAd.sT.GM vs.				Notes
		Buffer	rAd.null	rAd.sT	rAd.GM	
Tumor volume progression	Two-way ANOVA	****	ns	***	ns	Day 21
		****	ns	****	*	Day 25
Tumor weight	One-way ANOVA	****	ns	ns	ns	Day 25
	t-test	****	ns	ns	ns	
Lung metastatic nodules	One-way ANOVA	ns	*	ns	ns	Day 25
	t-test	*	***	ns	ns	
Lung luminescense	One-way ANOVA	**	ns	ns	ns	
	t-test	***	*	ns	ns	

Table S2: Comparsions of treatment efficacy of rAd.sT.GM-ICIs combination to other treatments and the buffer group in the combination study on day 25. Significance is indicated as *= $p < 0.05$, **= $p < 0.01$, ***= $p < 0.001$, ****= $p < 0.0001$.

Measurement	Statistical analysis methods	rAd.sT.GM + P + C vs.						
		Buffer	rAd.sT.GM	anti-PD-1 (P)	anti-CTLA-4 (C)	P + C	rAd.sT.GM + P	rAd.sT.GM + C
Tumor volume ratio	One-way ANOVA	****	***	***	ns	ns	***	ns
	t-test	****	***	****	**	*	****	ns
Tumor weight	One-way ANOVA	ns	ns	*	ns	ns	ns	ns
	t-test	*	**	**	ns	ns	ns	ns
Lung metastatic nodules	One-way ANOVA	****	ns	***	ns	ns	ns	ns
	t-test	****	*	****	***	***	**	ns
Lung metastatic area	One-way ANOVA	****	*	**	ns	ns	ns	ns
	t-test	****	**	**	*	ns	ns	ns
Lung luminescense	One-way ANOVA	**	ns	ns	ns	ns	ns	ns
	t-test	*	ns	*	ns	ns	ns	ns
