# Supplementary material for: Oncolytic adenovirus inhibits TNBC tumor growth/metastasis in mice by targeting TGF-β and overexpressing GM-CSF
Source: Mol Ther Oncol. 2025 Jan 17;33(1):200936. doi: 10.1016/j.omton.2025.200936 (PMC11919423; doi:10.1016/j.omton.2025.200936)
Supplement: Document S2. Article plus supplemental information [file mmc2.pdf]

Molecular Therapy
Oncology
Original Article

Oncolytic adenovirus inhibits TNBC tumor growth/metastasis in mice by targeting TGF-β and overexpressing GM-CSF

Nguyễn Thị Thanh Nhàn,[1,5] Soon Cheon Shin,[1,5] Beniamin Filimon,[1,5] Yuefeng Yang,[1,2] Zebin Hu,[1,3] Bruce Brockstein,[4] and Weidong Xu[1]

[1]Cancer Gene Therapy Program, Department of Medicine, NorthShore University HealthSystem, an Academic Affiliate of the University of Chicago Pritzker School of Medicine, Endeavor Health Research Institute, Evanston, IL, USA; [2]Beijing Jingda Biotechnology Co., Ltd., Beijing, China; [3]National Institutes for Food and Drug Control, Beijing, China; [4]Division of Hematology/Oncology, Kellogg Cancer Center, Northshore University Health System, Endeavor Health Medical Group, Evanston, IL, USA

Despite therapeutic advancements, metastatic triple-negative breast cancer (TNBC) remains mostly incurable and is a frequent cause of cancer-related deaths. We tested the hypothesis that inhibiting suppressive signals sustained by transforming growth factor (TGF)-β and concurrently stimulating recruitment of inflammatory cells with granulocyte-macrophage colony-stimulating factor (GM-CSF) by oncolytic viruses would result in improved anti-tumor responses. Thus, we developed a new oncolytic adenovirus rAd.sT.GM (AMUN-003) that expresses both sTGFβRIIFc (a TGF-β decoy), and GM-CSF and tested it in a mouse TNBC (4T1) subcutaneous model. rAd.sT.GM was safe to use and more effective in controlling tumor progression and lung metastasis following intratumoral injections when compared with control adenoviruses without modifications. In the same model, combinations of immune checkpoint inhibitor (ICI) therapy with rAd.sT.GM resulted in better inhibition of tumor growth and metastasis. Furthermore, we examined key immune response and prognosis biomarkers in sera, lungs, spleens, and tumors to evaluate the treatment efficacy. We found several key anti-tumor Th1 cytokines such as interleukin (IL)-2, IL-4, and interferon-γ, were stimulated by the combination therapy either systemically or in tumors or both, as well as anti-tumor biomarkers such as Granzyme B and perforin. These results support advancement to clinical testing with the combination therapy of rAd.sT.GM and ICIs for TNBC patients.

INTRODUCTION

Triple-negative breast cancer (TNBC) is a highly aggressive subtype of breast cancer with limited treatment options. Immune checkpoint inhibitors (ICIs) are effective in treating some breast cancers but less successful against immunosuppressive types like TNBC, underscoring the need for new therapies.[1,2]

Oncolytic viruses (OVs) selectively target and destroy cancer cells while sparing healthy tissues, presenting a promising solution for cancer treatment.[3–11] Among them, oncolytic adenoviruses (OAVs) offer unique advantages due to their well-characterized genomes, potential for extensive modifications to introduce new therapeutic targets, and established clinical safety.[12–14] Combining ICIs with OAVs has shown considerable potential in enhancing immune responses, inhibiting tumor growth, and reducing metastasis in several pre-clinical studies in different types of cancers such as colon cancer, pancreatic cancer, melanoma, and TNBC.[15–17]

Recent studies identify transforming growth factor (TGF)-β signaling as crucial in tumor immune evasion and resistance to ICIs.[18,19] Blocking TGF-β disrupts these processes and inhibits tumor progression in animal models.[20–22] We previously reported an OAV expressing soluble TGF-β receptor II and human immunoglobulin (Ig)G Fc fragment (sTGFβRIIFc) inhibited tumor-promoting signals and activated immune responses in mouse TNBC models.[23] On top of that, combining the OAV expressing sTGFβRIIFc with the tumor-homing cell-penetrating peptide LyP-1 and ICIs further suppressed tumor growth and metastasis.[24]

Granulocyte-macrophage colony-stimulating factor (GM-CSF) enhances anti-tumor immunity by improving dendritic cell function and activating T cells. GM-CSF has been used in various clinical settings for treatment purposes,[25] including acute myeloid leukemia (AML),[26] and non-small cell lung carcinoma.[27] The synergy between TGF-β inhibition and GM-CSF stimulation has been supported by various studies in murine models showing improved therapeutic outcomes when these pathways are targeted together.[28,29]

Received 12 July 2024; accepted 14 January 2025;
https://doi.org/10.1016/j.omton.2025.200936.

[5]These authors contributed equally

Correspondence: Weidong Xu, Cancer Gene Therapy Program, Department of Medicine, NorthShore University HealthSystem, an Academic Affiliate of the University of Chicago Pritzker School of Medicine, Endeavor Health Research Institute, 2650 Ridge Avenue, SB630, Evanston, IL 60201, USA.
E-mail: wxu@northshore.org

© 2025 The Author(s).
Published by Elsevier Inc. on behalf of The American Society of Gene and Cell Therapy.
This is an open access article under the CC BY license (http://creativecommons.org/licenses/by/4.0/).

To enhance our OAV's efficacy, we developed a novel adenoviral construct that combines TGF-β pathway disruption with GM-CSF's immunostimulatory effects and named it Ad.sT.GM (AMUN-003). This dual-expression-adenovirus targets TNBC cells, inhibits tumor-promoting signals, and stimulates immune responses, offering a potent strategy against this challenging cancer type.

RESULTS

Replication, cytotoxicity, and target protein expression of adenoviruses in breast cancer cell lines

The adenoviral backbone of rAd.sT.GM is from the AdEasy Adenoviral Vector System, which is rendered replication-defective by the deletion of the E1 and E3 genes that are essential for viral replication and modulation of the immune response, respectively. To make a new vector conditionally replicable in tumor cells, a telomerase reverse transcriptase promoter (TERTp) is used to control the reinstated viral replication E1A gene by exploiting the elevated telomerase activity in tumor cells but that is absent in normal post mitotic cells.[10,30–35] rAd.sT.GM also incorporates a dual expression system[11,36] whereby sTGFβRIIFc is governed by the CMV promoter, and the production of GM-CSF is driven by the internal ribosome entry site (IRES), enhancing the selectivity and therapeutic impact of the application (Figure S1).

We first assessed virus replication dynamics, cancer cell cytotoxicity, and target protein production in three breast cancer cell lines: MDA-MB-231 (a human TNBC cell line), MCF-7 (a human estrogen-dependent breast cancer cell line), and 4T1 (a murine TNBC cell line). The adenoviral vectors utilized in this study included Ad(E−).null (non-replicating control adenovirus without any therapeutic gene modification), rAd.null (replicating adenovirus without any therapeutic gene modification), rAd.sT (adenovirus expressing sTGFβRIIFc on the backbone of rAd.null), rAd.GM (adenovirus expressing GM-CSF on the backbone of rAd.null), and rAd.sT.GM (adenovirus co-expressing sTGFβRIIFc and GM-CSF).

Viral replication was quantified by assessing the viral burst size, which indicates the number of new virions produced per infected cell. Significant increases in viral replication were observed in cancer cells infected with the replicating adenoviral vectors (rAd.null, rAd.sT, rAd.GM, and rAd.sT.GM) compared with the non-replicating Ad(E−).null control in all cell lines we tested (Figures 1A–1C, the left panels). The replication ratios for all replicating adenoviral vectors in murine 4T1 cells were much lower than those in human cell lines (MDA-MB-231 and MCF-7) (ratios of less than 10-fold vs. those of around 10^3-fold). This disparity is anticipated, as the adenoviral vectors used are derived from human adenovirus serotype 5 (Ad5), which replicates more efficiently in human cell lines than in murine cell lines.

Cytotoxicity was quantified by determining the viral dose required to achieve 50% cell viability reduction (IC50). The replicating adenoviral vectors exhibited similar cytotoxic effects across the cell lines from both humans and mice (Figures 1A–1C, second panels from the left), suggesting even 4T1 cells do not support viral replication as efficaciously as human cell lines, yet they are still susceptible to toxic exposures. This finding confirms the cancer cell cytotoxicity of our adenoviral vectors.

The expression of the target proteins sTGFβRIIFc and GM-CSF was evaluated in the infected cancer cell lines by ELISA assay. As expected, secreted sTGFβRIIFc was detected only in cells infected with rAd.sT and rAd.sT.GM, while GM-CSF was detected exclusively in cells infected with rAd.GM and rAd.sT.GM (Figures 1A–1C, the third and fourth panels). These results confirm that sTGFβRIIFc, GM-CSF, or both can be expressed after the infection of corresponding adenoviral vectors effectively in MDA-MB-231, MCF-7, and 4T1 cell lines.

Safety and systemic pharmacokinetics of replicating adenoviruses in the 4T1 mouse model

We further investigated the safety and efficacy of these adenoviruses via intratumoral administration in a 4T1 mouse model. Tumor cell inoculation and treatment strategies are described in the corresponding materials and methods section and in Figure S2. Serum levels of lactate dehydrogenase (LDH), aspartate aminotransferase (AST), and alanine transaminase (ALT), markers of systemic toxicity and hepatotoxicity, respectively, were assessed 24 h after adenovirus administration (day 10), showing no significant changes in treated animals compared with controls (Figures 2A–2C). Serum concentrations of pro-inflammatory cytokines interleukin (IL)-6, tumor necrosis factor (TNF)-α, and IL-1β were also measured, with no notable increases observed (Figures 2D–2F). These results suggest a favorable safety profile of our OAVs.

The efficacy of systemic target gene expression was determined by quantifying serum levels of sTGFβRIIFc, confirming its presence in animals treated with rAd.sT and rAd.sT.GM (Figure 2G). Notably, changes in blood sTGFβRIIFc expression were also detectable for both rAd.sT and rAd.sT.GM on day 25 post tumor cell inoculation, suggesting sTGFβRIIFc is systemically active even at this late time point. However, we did not find detectable levels of human GM-CSF expression in all blood samples (data not shown), which is consistent with the previous pharmacokinetics study of serum human GM-CSF levels in the mouse models.[37–39] Blood TGFβ-1 expression levels were also measured at both day 10 and day 25 (Figure 2H). TGFβ-1 expression levels remained unaltered on day 10, indicating that the treatments in the initial phase did not impact TGFβ-1 signaling effectively. However, by day 25, a significant decrease in TGFβ-1 expression was observed in the rAd.sT- or rAd.sT.GM-treated group, suggesting that existence of prolonged treatment effects in the inhibition of the TGFβ-1 signaling pathway systemically.

Local efficacy and inhibition of tumor growth and metastasis by intratumoral injection of rAd.sT.GM in the 4T1 mouse model

To further demonstrate the efficacy and oncolytic functions of the adenoviruses, we evaluated changes of key targets in the tumor

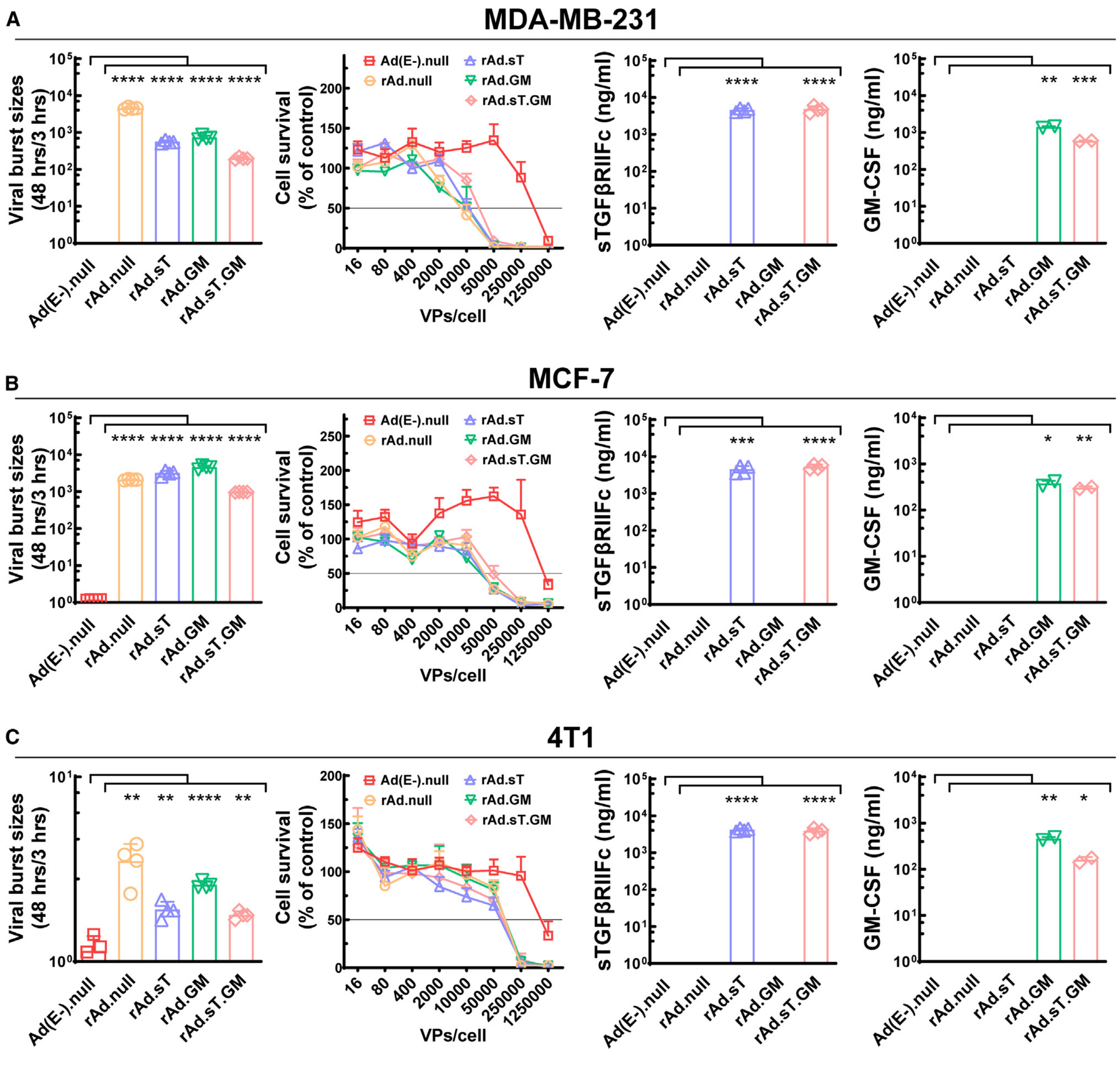

Figure 1. Adenovirus replication, cell survival, and sTGFβRIIFc or GM-CSF expression in infected breast cancer cells

(A) MDA-MB-231 cells were infected with Ad(E−).null, rAd.null, rAd.sT, rAd.GM, and rAd.sT.GM (AMUN-003), respectively, and data of adenovirus replication, cell survival, and sTGFβRIIFc or GM-CSF expression are shown. (B) Shown are adenovirus replication, cell survival, and sTGFβRIIFc or GM-CSF expression for infected MCF-7 cells. (C) Shown are adenovirus replication, cell survival, and sTGFβRIIFc or GM-CSF expression for infected 4T1 cells. Significant difference (compared with Ad(E−).null by unpaired t tests) is shown as *$p < 0.05$, **$p < 0.01$, ***$p < 0.001$, ****$p < 0.0001$.

samples and the abilities of adenoviral vectors to inhibit tumor growth and metastasis during the course of this study. First, the tumor expression of viral genomes, sTGFβRIIFc, and human GM-CSF was analyzed in tumor samples collected 24 h after the second adenovirus injection (day 10) and at the end of the experiment (day 25) by qRT-PCR. Increased viral genome DNA expression was detected in tumors with adenoviral vector injection, although only that of the rAd.sT, rAd.GM or rAd.sT.GM group was significantly higher compared with the untreated control when the Mann-Whitney *U* test was applied for these non-parametric data (Figure 3A). No significant changes of viral genome DNA expression were detected between any groups on day 25, suggesting no virus was still present at this late time point. sTGFβRIIFc expression levels were significantly increased in the tumor tissues of rAd.sT

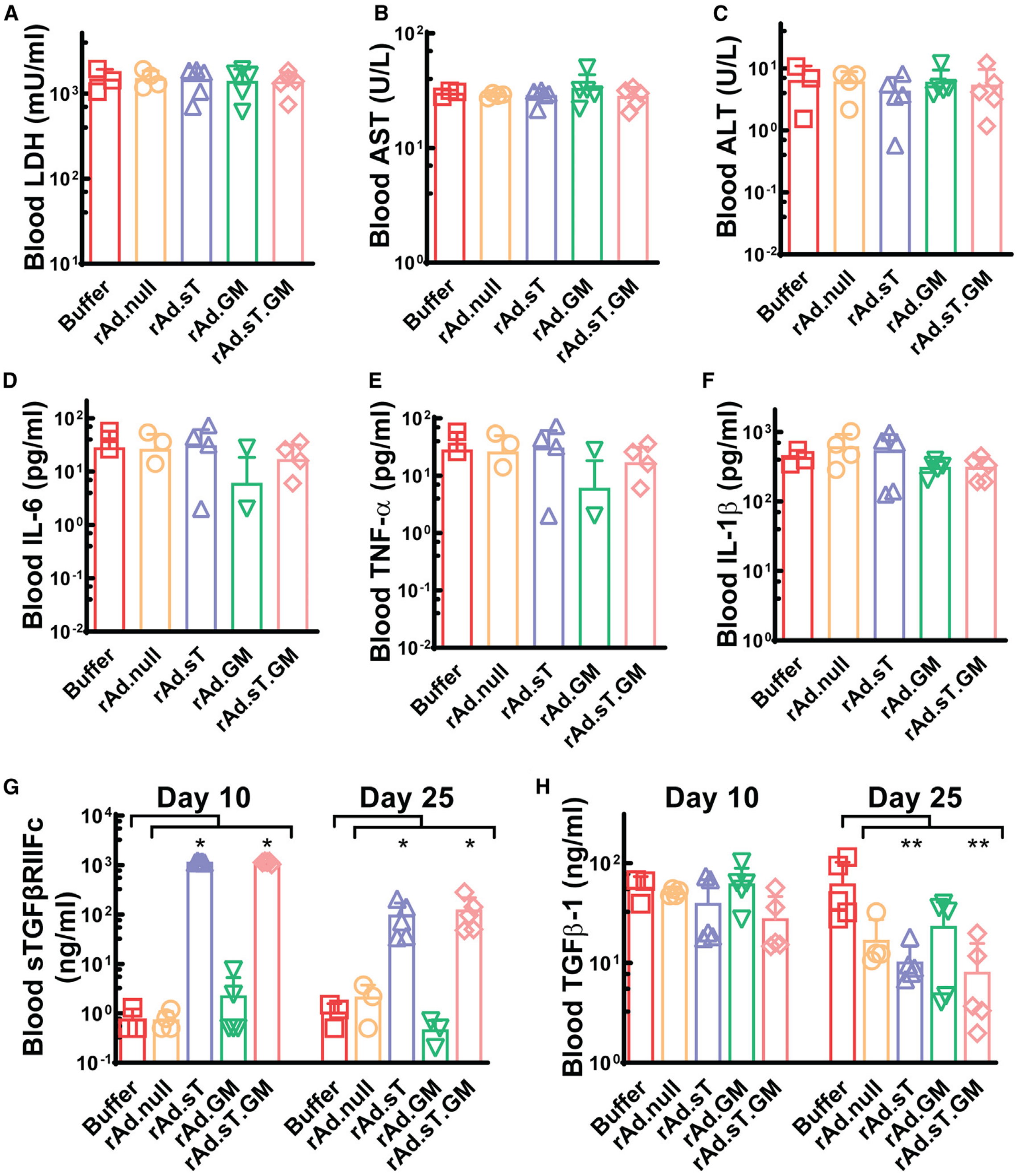

Figure 2. Safety and systemic pharmacokinetics analysis of intratumoral administration of replicating adenoviruses in the 4T1 mouse model

(A–C) Serum LDH, AST, and ALT levels in mouse sera are shown, respectively. (D–F) Levels of three pro-inflammatory cytokines, IL-6, TNF-α, and IL-1β in serum were measured and are shown, respectively. (G) Shown are serum sTGFβRIIFc levels on both day 10 and day 25. (H) Serum TGFβ-1 levels on both day 10 and day 25 are shown. Significant difference, compared with the buffer group by Mann-Whitney *U* tests, is shown as *$p < 0.05$ and **$p < 0.01$.

and rAd.sT.GM groups when compared with the buffer-treated group on day 10, and were still increased in the rAd.sT.GM group when compared with the Ad.null group on day 25 (Figure 3B). Although treatments with rAd.GM and rAd.sT.GM led to detectable GM-CSF expression in tumors on day 10, the increase was statistically significant for rAd.sT.GM alone by the Mann-Whitney *U* test (Figure 3C). Unlike serum human GM-CSF, human GM-CSF expression was still detectable in the rAd.GM and rAd.sT.GM group on day 25, and both groups had significantly higher GM-CSF expression levels when compared with the rAd.sT group. These

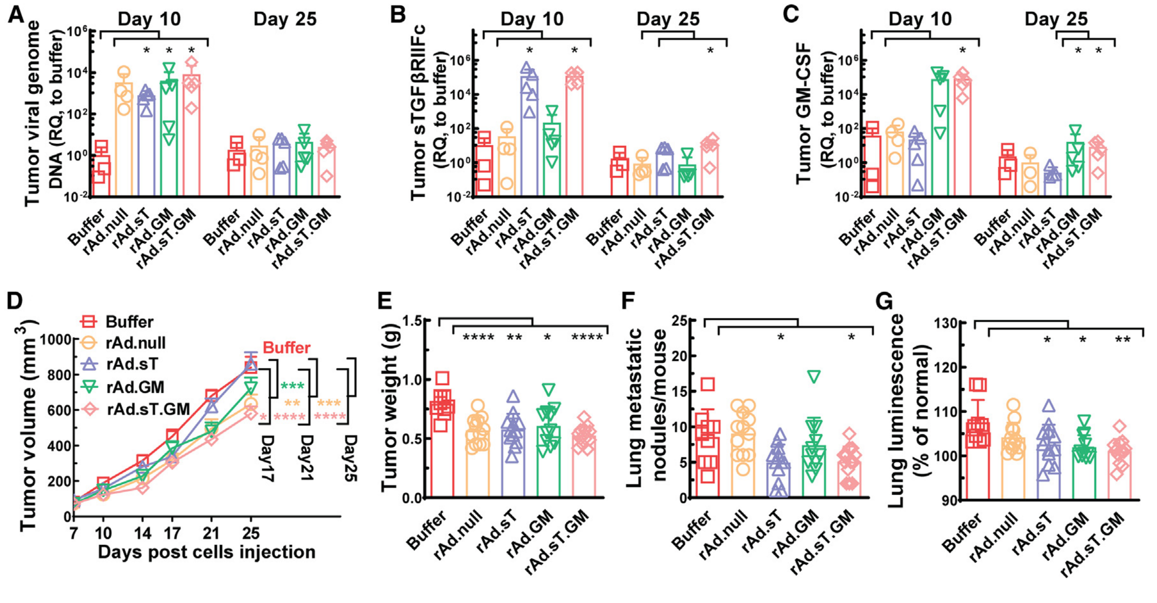

Figure 3. Local efficacy and inhibition of tumor growth and metastasis by intratumoral injection of replicating adenoviruses in the 4T1 mouse model

(A–C) Tumor expression of viral genome, sTGFβRIIFc, and GM-CSF were measured by qRT-PCR for both day 10 and day 25 samples, and are shown, respectively. (D) The primary 4T1 tumor growth was measured by tumor volume for 25 days and is shown. (E) Shown are weights of primary tumors at the terminal point (day 25). (F) Lung surface metastatic nodules for each mouse were counted and are shown. (G) Shown are lung luminescence from metastatic 4T1-luc2 cells measured by luciferase assay. Mann-Whitney *U* tests were used for significant difference analysis for (A), (B), and (C); two-way ANOVA with Tukey's multiple comparisons were used for primary tumor progression analysis over time in (D). One-way ANOVA with Bonferroni's multiple comparisons tests were used for group statistical analyses of (E) and (G); unpaired t tests were used to compare each treatment group with the buffer in (F). Significant differences to the buffer group or other indicated groups are shown as $^{*}p < 0.05$, $^{**}p < 0.01$, $^{***}p < 0.001$, or $^{****}p < 0.0001$.

findings confirm the efficacy of viral infection and target protein expression after intratumoral administration of our adenoviruses in the 4T1 mouse model.

Effective analysis revealed that all adenovirus treatments significantly inhibited tumor growth, as evidenced by either tumor progression analysis by tumor volume measurements or tumor weight analysis (Figures 3D and 3E). rAd.sT.GM is superior to other vectors in inhibiting primary tumor growth since it is the only group that had significant reduction of tumor volume on day 17 as well as day 21 and day 25 when compared with the untreated group (Figure 3D). Additionally, only rAd.sT and rAd.sT.GM treatments significantly inhibited the appearance of lung surface metastatic nodules (Figure 3F). Furthermore, rAd.sT, rAd.GM, and rAd.sT.GM treatments effectively inhibited metastatic 4T1-luc2 cells in the lungs, as demonstrated by lung luminescence analysis (Figure 3G). Notably, while the rAd.null treatment was effective in inhibiting tumor growth (Figures 3D and 3E), it did not effectively prevent lung metastasis (Figures 3F and 3G). Indeed, rAd.sT.GM is the best group for treatment by both tumor size and lung metstasis analysis as shown in Table S1 since the rAd.sT.GM treatment led to favorable responses when compared with other treatment options too. These results underscore the crucial role of sTGFβRIIFc and GM-CSF co-expression by rAd.sT.GM in achieving better inhibition of both tumor growth and lung metastasis.

Combination treatment of rAd.sT.GM with anti-PD-1 and/or anti-CTLA-4 antibodies remarkably inhibited primary tumor growth and spontaneous lung metastasis in the 4T1 mouse model

Given that rAd.sT.GM consistently inhibits both tumor growth and metastasis, we proceeded to test its efficacy in combination with anti-PD-1 (P) and anti-CTLA-4 (C) antibodies. For this set of experiments, we reduced the total doses of adenoviral vector to 5.0×10^{10} VPs/mouse to lessen the potential risks of toxicity with combinations. We found that the rAd.sT.GM + anti-CTLA-4 group and the triple treatment group (rAd.sT.GM + anti-PD-1 + anti-CTLA-4) are the best groups for inhibiting tumor volume during the course of the study (Figure 4A) with direct statistical comparisons of them with the buffer and other treatments shown in the table in Figure 4A. Also, on day 25, when compared with the anti-PD-1 + anti-CTLA-4 (P + C) group, only the triple treatment is significant in inhibiting tumor volume. Tumor growth ratio (day 25 tumor volume vs. day 10 tumor volume) analysis further showed a significant reduction of tumor progression in both the rAd.sT.GM + anti-CTLA-4 group, and the triple treatment group compared with the control group (Figure 4B). By tumor weight analysis at day 25, significant inhibition could be detected in comparison with the rAd.sT.GM + anti-CTLA-4 treatment to the untreated, rAd.sT.GM, anti-PD-1, and anti-CTLA-4, respectively. However, for the triple treatment group we only detected significant changes with the anti-PD-1 treatment, suggesting further analysis in metastasis is needed to fully understand their efficacy (Figure 4C).

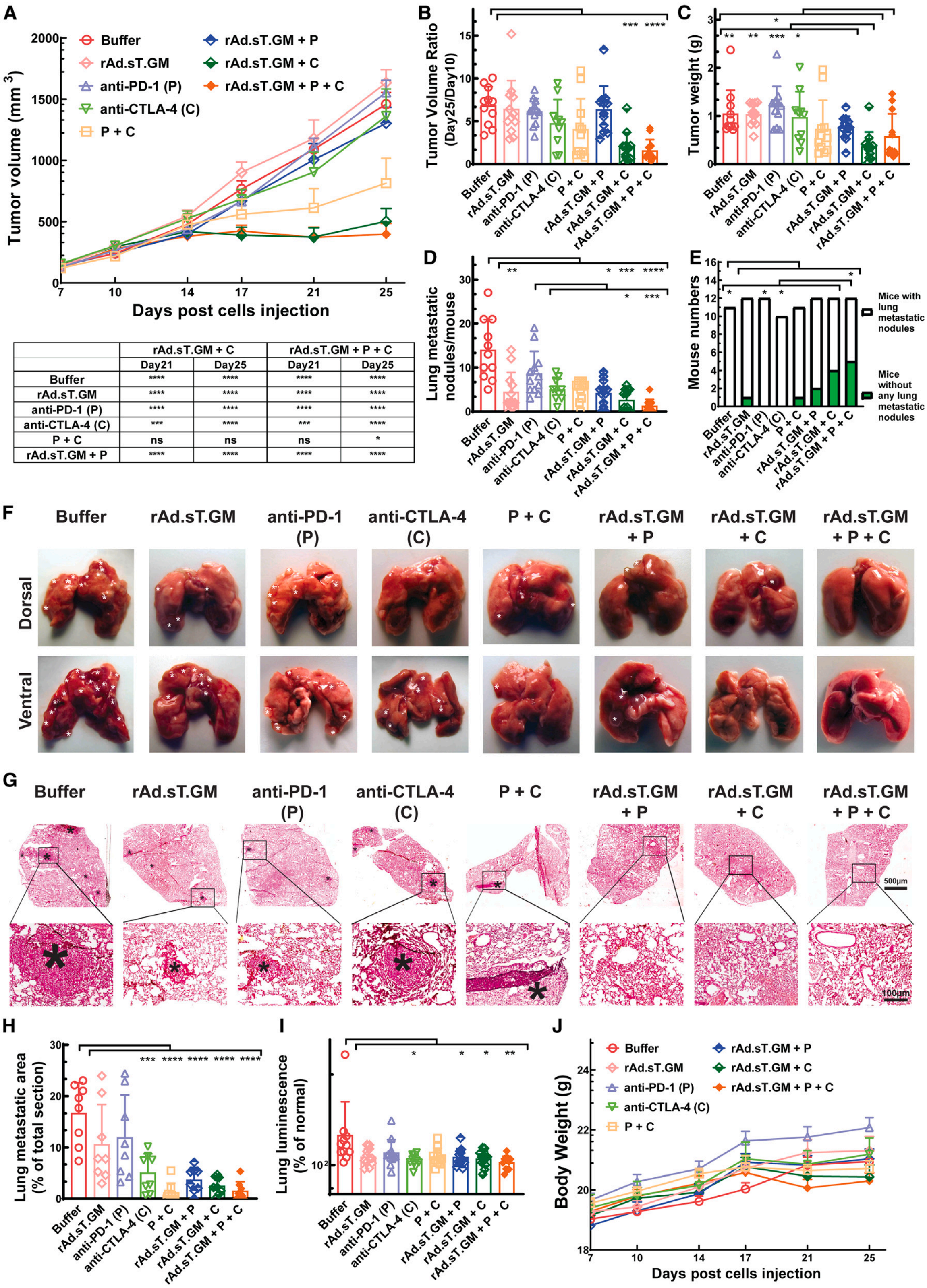

	rAd.sT.GM + C		rAd.sT.GM + P + C	
	Day21	Day25	Day21	Day25
Buffer	****	****	****	****
rAd.sT.GM	****	****	****	****
anti-PD-1 (P)	****	****	****	****
anti-CTLA-4 (C)	***	****	***	****
P + C	ns	ns	ns	*
rAd.sT.GM + P	****	****	****	****

(legend on next page)

(legend on next page)

For the metastasis study, we focused on the lung metastasis analysis by counting visible lung macrometastasis numbers on the lung surface, quantifying both micrometastasis and macrometastasis area in sectioned lung tissues, and measuring lung luminescence from migrating 4T1-luc cells in tissue lysis on samples we obtained on day 25. Lung surface macrometastatic nodules were significantly reduced by rAd.sT.GM treatment and all rAd.sT.GM and ICI combinations compared with the untreated group (Figure 4D). Interestingly, none of the treatments with ICIs (anti-PD-1, anti-CTLA-4, or anti-PD-1 + anti-CTLA-4) alone was able to significantly decrease lung metastatic nodules compared with the untreated. The triple treatment may be the best group to inhibit lung metastatic nodule appearance since when compared with the anti-PD-1 treatment the p value is less than 0.001 but for the rAd.sT.GM + anti-CTLA-4 treatment it is less than 0.05. Notably, when counting the number of mice with and without lung metastasis nodules, only the triple treatment group showed a significant reduction in the number of mice with metastasis nodules compared with the untreated group (Figure 4E). The presentative images of lung surface macrometastatic nodules are shown in Figure 4F. H&E-stained lung sections revealed both micrometastases and macrometastases (Figure 4G), with anti-CTLA-4, anti-PD-1 + anti-CTLA-4, rAd.sT.GM + anti-PD-1, rAd.sT.GM + anti-CTLA-4, and the triple treatment all having significantly reduced metastatic burden compared with the untreated (Figure 4H). For the lung luminescence analysis, significant inhibition was detected for the anti-CTLA-4, rAd.sT.GM + anti-PD-1, rAd.sT.GM + anti-CTLA-4, and the triple treatment group compared with the untreated (Figure 4I). To summarize, a supplementary table with comparsions of rAd.sT.GM-ICIs combination to other treatments and the buffer group is provided (Table S2). Taken together, the ICI treatments combined with rAd.sT.GM generally are more consistent in inhibiting lung metastasis by our analysis.

Throughout the experiment, mouse body weights were monitored, showing no significant deviations (Figure 4J). To be noted, the reduced dose of rAd.sT.GM alone did not significantly alter tumor volume but it still inhibited lung metastasis (Figure 4D). Importantly, when compared with groups without rAd.sT.GM injection, rAd.sT.GM and ICI combinations demonstrated significant improvements, suggesting the importance of rAd.sT.GM in priming anti-tumor immune responses for immune checkpoint-based therapies.

The combination treatments of rAd.sT.GM with anti-CTLA-4 and anti-PD-1 antibodies changed the cytokine profile favoring anti-tumor immunity

We then tested the effects of treatments on the cytokine profile to understand the underlying mechanism to better guide us for clinical translations. We tested TGFβ-1 expression levels in mouse serum for all treatment groups by TGFβ ELISA as described in the materials and methods section and found it was significantly reduced by rAd.sT.GM, anti-PD-1 + anti-CTLA-4, and the triple treatment (Figure 5A). An MSD V-PLEX Mouse Cytokine 19-Plex Kit was used to quantify the levels of interferon (IFN)-γ, IL-1β, IL-2, IL-4, IL-5, IL-6, IL-9, IL-10, IL-12p70, IL-15, IL-17A/F, IL-27p28/IL-30, IL-33, IP-10, KC/GRO, MCP-1, MIP-1α, MIP-2, and TNF-α in mouse sera from selected groups. Among them, we found significant reduction of IL-1β, IL-10, IL-30, and TNF-α by several treatment groups when compared with the untreated (Figures 5B–5E). The triple treatment led to reduced levels of all four cytokines, but only its reduction in IL-1β and TNF-α was significant compared with the untreated. Given the excellent efficacy of the triple treatment in inhibiting tumor progression, it is not surprising to us since TGFβ-1, IL-1β, and IL-10, and IL-30 are key mouse Th2, Th17, and Treg differentiation cytokines that have been linked to immune suppression during cancer development. On the contrary, we detected a significant increase in several key Th1 cytokines including IFN-γ, IL-2, and IL-15 (Figures 5F–5H) by the triple treatment, and all of them are crucial for anti-tumor immunity by enhancing the activity of cytotoxic T cells and natural killer cells. To be noted, the level of TNF-α, another Th1 cytokine, was significantly reduced in the triple treatment group. Since TNF-α can exhibit both anti-tumor activities by inducing apoptosis and necrosis in tumor cells[40–42] and pro-tumoral activities by regulating immune cells,[43,44] we speculate that the inhibition of it by the triple treatment would suppress the pro-tumoral activity of TNF-α. For other treatment groups, their cytokine profile is less closely associated with anti-tumor immune responses. For example, the rAd.sT.GM + anti-CTLA-4 treatment, which also impressively inhibited tumor progression and metastasis as shown early, only significantly reduced the production of IL-1β, IL-10, and IL-30 that was related to immunosuppression, but did not increase the expression levels of any stimulating Th1 cytokine we tested. In general, our cytokine profiling results indicated a shift toward a more anti-tumor cytokine profile, especially by the triple treatment, supporting the efficacy of this

Figure 4. Combinations of rAd.sT.GM (AMUN-003) with anti-CTLA-4 and anti-PD-1 antibodies (the triple treatment) most significantly inhibited both primary tumor growth and spontaneous lung metastasis in the 4T1 mouse model

(A) Shown are primary 4T1 tumor progression for 25 days measured by tumor volume and statistical analysis of all other groups compared with rAd.sT.GM + anti-CTLA-4 or the triple treatment (rAd.sT.GM + P + C) on day 21 and day 25. (B) Tumor growth ratios (day 25 tumor volume vs. day 10 tumor volume) were measured and are shown. (C) Shown are tumor weights at the terminal point (day 25) for all groups. (D) Lung surface metastatic nodules for each mouse were counted and shown. (E) The numbers of mice with or without lung surface metastatic nodules for each group were counted and statistical analysis is shown. (F) Representative images of the gross appearances of lungs at the end of the study. (G) Representative images of H&E-stained lung sections of each group. (H) Shown is microscopic lung metastasis analysis for all groups. (I) Lung luminescence from metastatic 4T1-luc2 cells were measured and is shown. (J) Mouse body weights were measured through the experiment and are shown. * in (F) and (G) indicates the site of lung macrometastases and micrometastases. Magnification in (G) is indicated by scale bars (the scale bar for low magnification images is 500 μm; that for high magnification is 100 μm). Two-way ANOVA with Tukey's multiple comparisons were used for primary tumor progression analysis over time in (A). One-way ANOVA (Kruskal-Wallis or ordinary) with Dunn's or Bonferroni's multiple comparisons tests were used for group statistical analyses of (B), (C), (D), (H), and (I). Fisher's exact tests were used in (E) to determine significant differences between two categorical groups. Significant differences to the buffer group or other indicated groups are shown as *$p < 0.05$, **$p < 0.01$, ***$p < 0.001$, or ****$p < 0.0001$.

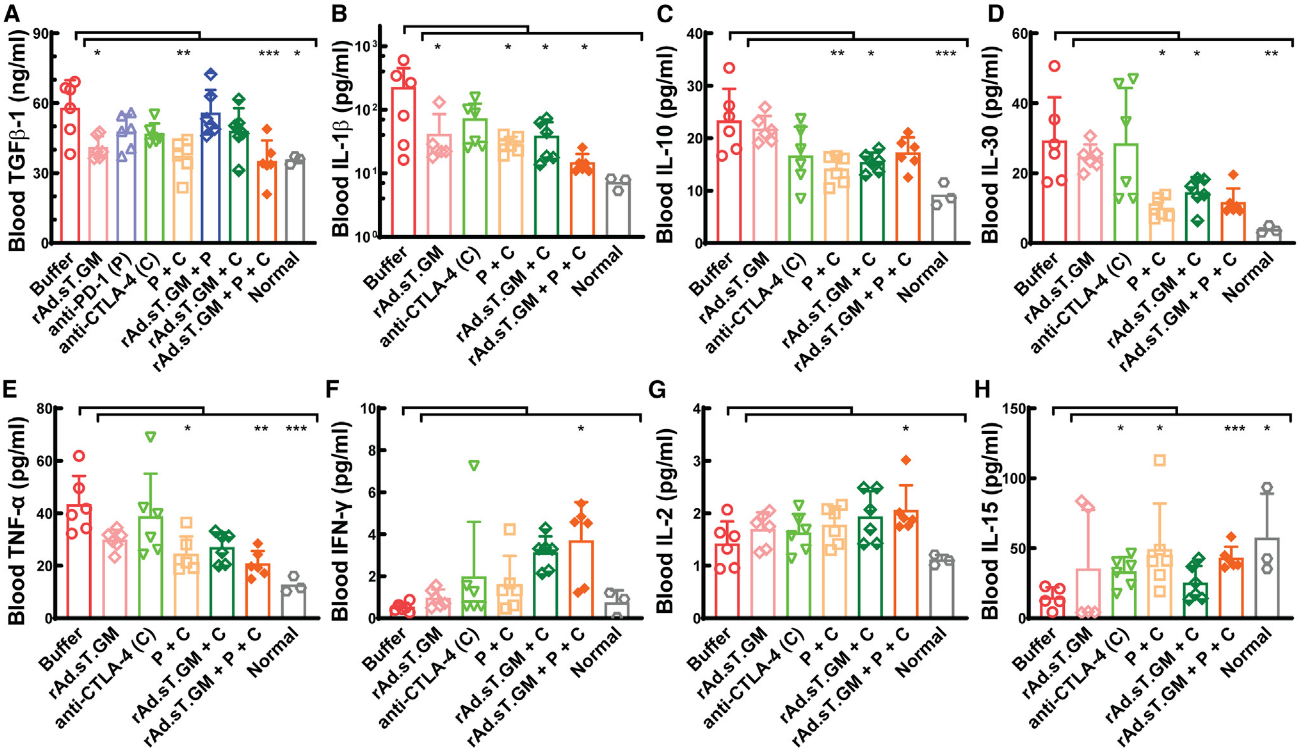

Figure 5. The triple treatment (rAd.sT.GM + P + C) led to more evident changes of cytokine profile favoring anti-tumor immunity on day 25

(A) Blood TGFβ-1 expression was measured for all groups by a TGFβ-1 ELISA kit and is shown. (B–H) Shown are blood IL-1β (B), IL-10 (C), IL-30 (D), TNF-α (E), IFN-γ (F), IL-2 (G), and IL-15 (H) levels measured by the MSD V-PLEX cytokine kits in selected groups. Ordinary one-way ANOVA with Bonferroni's multiple comparisons tests were used for group statistical analyses in (A–F); unpaired t tests were used to compare each treatment group with the buffer in (G) and (H). Significant differences to the buffer group are shown as *$p < 0.05$, **$p < 0.01$, ***$p < 0.001$, or ****$p < 0.0001$.

combination therapy in fostering a robust immune response against tumor cells.

The combination treatment of rAd.sT.GM with anti-CTLA-4 and anti-PD-1 antibodies stimulated anti-tumor responses in lung, spleen, and tumor tissues

Finally, the assessment of our treatments on anti-tumor responses was conducted via qRT-PCR analysis of several key targets and markers in lung, spleen, and tumor tissues. In the lung tissues, the triple treatment group was the only group showing a significant increase of Granzyme B, a critical component of the cytotoxic T cell response, suggestive of an enhanced anti-tumor immunity in lungs (Figure 6A).

Also, significant increases of several key cytokine expressions by the triple treatment were observed in the spleen, indicating activation of a robust systemic anti-tumor immunity. IL-2, IL-4, and IFN-γ all demonstrated significantly elevated expression by the triple treatment (Figures 6B–6D). In tumor tissues, expression levels of IL-4, IFN-γ, and perforin, an indicator of anti-tumor cytotoxicity, were significantly increased by several treatment groups, while the triple treatment significantly upregulated the expression of IFN-γ (Figures 6E–6G). The following are other treatment groups that exhibited stimulatory activities by our analysis: rAd.sT.GM and anti-PD-1, which both enhanced the expression of IL-4 and perforin in tumor; anti-CTLA-4, in which increases of spleen IL-2 and tumor IFN-γ and perforin were detected; anti-PD-1 + anti-CTLA-4, with enhanced tumor IFN-γ expression; rAd.sT.GM + anti-PD-1, in which spleen IL-2, IL-4, and IFN-γ expression and tumor IL-4 and perforin expression were upregulated; and rAd.sT.GM + anti-CTLA-4, in which all targets above were increased except for spleen IL-2 and IFN-γ. Clearly, the combination of rAd.sT.GM with any ICIs has a broader range of activation of these anti-tumor signaling pathways. Interestingly, TGFβ-1 exhibited differential expression patterns in the tumor and spleen tissues. In the tumor tissue, TGFβ-1 expressions were inhibited in all combination treatment groups when compared with the rAd.sT.GM group, suggesting a stronger blockade of TGFβ-1 mediated pro-tumorigenic signaling in the tumor microenvironment by the combinations (Figure 6H). Conversely, in the spleen, most of our treatments, except for rAd.sT.GM and anti-PD-1, led to significantly increased expression of TGFβ-1 compared with the buffer group, which may indicate a systemic activation of TGFβ-1 may still be required for the other functions of TGFβ-1 in this mouse model (Figure 6I). Information we learn from these local and systemic cytokine and biomarker analysis

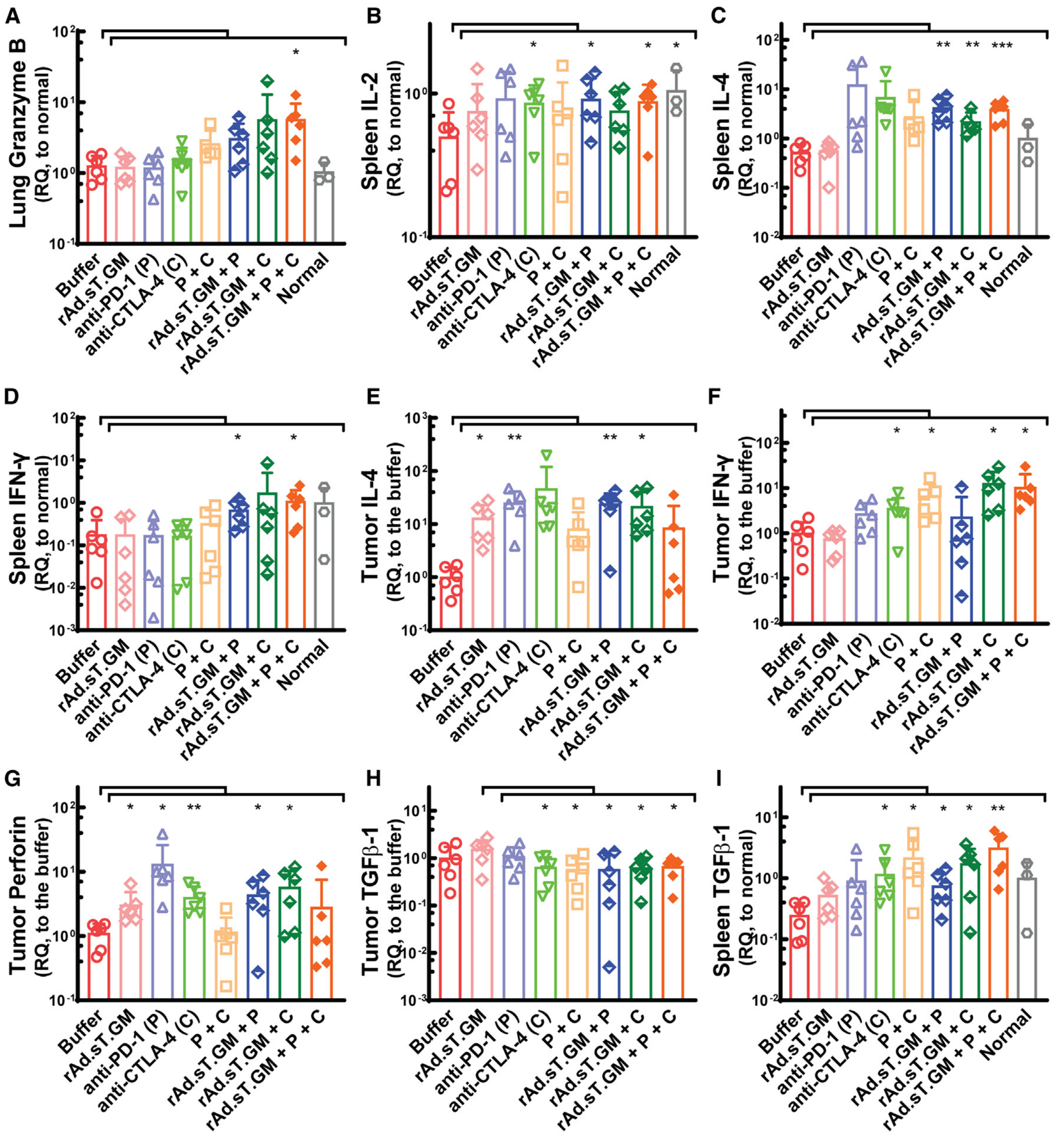

Figure 6. The combination treatments of rAd.sT.GM with ICIs have a broader range of anti-tumor immune activation in lung, spleen, and tumor tissues obtained on day 25 by qRT-PCR analysis

(A) Lung Granzyme B levels in all groups were measured and shown. (B–D) Shown are IL-2, IL-4, and IFN-γ expression in spleen, respectively. (E–G) Shown are IL-4, IFN-γ, and perforin expression in tumor. (H and I) Shown are TGFβ-1 expression levels in tumor and spleen tissues, respectively. Unpaired t tests were used to compare each treatment group to the buffer for all data. Significant difference to the buffer group is shown as *$p < 0.05$, **$p < 0.01$, or ***$p < 0.001$.

will help us to screen patients for personalized treatments in future clinical trials.

To move forward with this approach with investigational new drug (IND) filing and clinical trials, most recently we completed a survival analysis to confirm its efficacy (Figure S3). Our data showed, when compared with the buffer group, both rAd.sT.GM + anti-PD-1 + anti-CTLA-4 (rAd.sT.GM-ICIs) and rAd.sT.GM + anti-CTLA-4 groups have significant differences as $p = 0.0001$, which are best in all comparisons of treatments to the buffer.

Taken together, our results highlight the important role of rAd.sT.GM in priming effective anti-tumor immune responses in this model and suggest that the combination therapy with rAd.sT.GM

and ICIs is suitable to be tested further in clinical trials for TNBC treatment.

DISCUSSION

This study presents a significant advancement in the treatment of TNBC through the development of a novel oncolytic adenovirus (rAd.sT.GM, AMUN-003). Our findings indicate that rAd.sT.GM, which co-expresses sTGFβRIIFc and GM-CSF, effectively inhibits tumor growth and reduces metastasis in TNBC models, compared with other control adenoviruses which do not express sTGFβRIIFc and GM-CSF together, or any of them. The combination treatment strategy with ICIs, especially the triple treatment with both ICIs, not only is more effective in the inhibition of tumor growth and metastasis, but also disrupts tumor-promoting signals while stimulating anti-tumor immune responses, suggesting a promising approach for aggressive cancers like TNBC.

The dual targeting of TGF-β signaling and GM-CSF stimulation is promising in addressing the highly immunosuppressive nature of many TNBC patients that may not respond to current immunotherapy approaches. TGF-β signaling plays a pivotal role in promoting tumor growth and immune evasion by fostering a suppressive microenvironment that inhibits effective anti-tumor immune responses.[19,21] Simultaneously, GM-CSF enhances anti-tumor immunity by improving dendritic cell function and activating T cells. GM-CSF, when combined with other treatments such as immunotherapy, chemotherapy, or radiation therapy, has demonstrated effective outcomes in melanoma and other cancer patients.[25,45,46] Furthermore, the combination of TGF-β blockade and GM-CSF has been shown to enhance chemotherapy effects in pancreatic cancer models[29] and provides a survival advantage with GM-CSF-secreting allogeneic pancreas tumor vaccine GVAX.[28] The safety and efficacy of a TGF-β2 antisense and GM-CSF vaccine has also been tested in a phase I trial for a wide range of solid tumor patients.[47] Although many studies focus on this combination, it remains unknown if TGF-β blockade and GM-CSF are effective against TNBC's immunosuppressive nature. Here, we report for the first time that co-expressing sTGFβRIIFc and GM-CSF by OAVs demonstrates both the safety and efficacy of this combination in TNBC.

Our promising results of rAd.sT.GM in TNBC also suggest potential applications in other cancers characterized by aberrant TGF-β signaling and/or suppressive immune microenvironment, such as colon cancer,[48] ovarian cancer,[49] and glioblastoma.[50] Given the crucial role of TGF-β in immune resistance and direct immune-stimulatory functions of GM-CSF, targeting these signaling pathways could be effective in a broader range of tumors. Moreover, 4T1 tumors in this study are known to be notoriously non-immunogenic and difficult to treat, and our treatment strategy would be potentially potent for other resistant and aggressive cancer types too.

The integration of OVs with ICIs represents a rapidly expanding field with significant therapeutic potential. OVs prime the tumor microenvironment for immune attack by lysing tumor cells and releasing tumor antigens, which in turn enhances the efficacy of ICIs that block inhibitory signals and activate anti-tumor immune responses.[6,20] This combination has shown remarkable results in various cancers and is the subject of numerous clinical trials exploring its efficacy. For example, the combination of anti-PD-1 and anti-CTLA-4 with OAV expressing GM-CSF inhibited tumor growth and prolonged survival in a TNBC mouse model.[17] A phase I/II study of Pexa-Vec OV in combination with durvalumab, an anti-PD-L1 antibody, showed positive activity in patients with advanced colorectal cancer.[51] Our study contributes to this growing field by demonstrating that rAd.sT.GM, when combined with ICIs, significantly improves outcomes in TNBC models, further validating the potential of this therapeutic strategy. It is noteworthy that for this highly aggressive 4T1 mouse model the low dose of rAd.sT.GM alone, as shown in the combination therapy experiments, did not effectively inhibit primary tumor growth. These results were expected since our time course study showed that blood sTGFβRIIFc expression on day 25, although it was still detectable with a higher dose, was much lower than that on day 10 (Figure 3C). Thus, for the treatment of other aggressive cancers, combination with other immunotherapy approaches would be the key for more successful drug development.

Mouse tumor models have proved valuable for pivotal pharmacology studies to enable a successful Investigational New Drug (IND) filing to start clinical trials. Practically, with the 4T1 TNBC model that we used in the study, we were able to assess the effectiveness of different treatment combinations, and the roles of the immune system in response to our treatments. However, since the replication of human Ad5 adenoviruses is not supported by mouse cells, we have not obtained the proper exposure and toxicity data to support first-in-human (FIH) dosing. Our collaborating teams with the early-stage biotechnology company, Amunbio, have been working with other animal models and human tissues to ensure that all safety measures are in place and implemented in the future. To be noted, rAd.sT.GM is a conditionally replicating OAV that preferentially replicates within cells with elevated telomerase activity, mostly cancer cells, and subsequently kills them. Currently, many conditionally replicating OAVs have been proved safe for clinical trials[52,53] and recently the Food and Drug Administration has granted both fast track and breakthrough therapy designations to cretostimogene grenadenorepvec (CG0070), a conditionally replicating OAV targeting Rb-defective cancer cells for use as a prospective treatment in patients with high-risk BCG-unresponsive non-muscle invasive bladder cancer.[54,55]

In conclusion, the study reported here highlights the effectiveness of combining TGF-β blockade with GM-CSF stimulation via OAV and ICI treatment in the 4T1 mouse model. This approach offers a powerful strategy against TNBC and potentially other cancers. Future research should focus on further mechanistic studies and clinical trials to validate these findings and expand their therapeutic applications for other resistant cancers and/or in clinical trials.

MATERIALS AND METHODS

Cell culture

Human breast cancer cell lines, MDA-MB-231 and MCF-7, and mouse breast cancer cell line, 4T1, 4T1-luc (ATCC, Manassas, VA), were

purchased and maintained as described earlier.[10,12,20,23,24] ATCC STR profiling tests for cell line authentication were recently performed for 4T1 with a 98% match of the database profile of the ATCC 4T1 cell line (CRL-2539) (ATCC STR profiling test FTA Barcode: MUSA3575; Sales Order: SO2111801) and 4T1-luc with the result of 100% match of the database profile of the 4T1 cell line (CRL-2539) (ATCC STR profiling test FTA Barcode: MUSA3576, Sales Order: SO2111801). They were also previously MAP (IMPACT by PCR method) tested in the Research Animal Diagnostic Laboratory, University of Missouri, Columbia, MO, and found to be free of *Mycoplasma* spp, Sendai virus, Mouse hepatitis virus, Pneumonia virus of mice, Minute virus of mice, Mouse parvovirus (MPV1, MPV2, MPV3), Theiler's murine encephalomyelitis virus, Murine norovirus, Reovirus 3, Mouse rotavirus, Ectromelia virus, Lymphocytic choriomeningitis virus, Polyoma virus, and lactate dehydrogenase-elevating virus.

Adenoviruses

Recombinant oncolytic adenoviruses rAd.sT, rAd.GM, and rAd.sT.GM, expressing sTGFβRIIFc, GM-CSF, and both proteins, respectively, were developed using a simplified system as described previously.[10,11,31,56,57] These viruses used the backbone from the AdEasy Adenoviral Vector System, rendered replication-defective by deleting the E1 and E3 genes. To enable selective replication in tumor cells, the TERT promoter (TERTp) is used to regulate E1A gene expression. Additionally, rAd.null, an oncolytic adenovirus regulated by TERTp without therapeutic gene modifications, and the non-replicating Ad(E1-).null were also produced using the AdEasy System (Figure S1). Good laboratory practice (GLP) mass productions of viruses for animal studies were prepared by Gene Vector Core of Baylor College of Medicine (Houston, TX). They were tested negative for endotoxin and sterility.

Adenoviral replication assay

MDA-MB-231, MCF-7 cells, or 4T1-luc cells were seeded into six-well plates at a density of 2.5×10^5 cells per well. The next day, cells were infected with adenoviral vectors (2.5×10^5 viral particles [VPs] per cell for a total of 5.0×10^5 cells each well) for 3 h. After washing all cells three times with PBS and cell culture media, the crude viral lysates were collected with media immediately for 3 h samples, or at 48 h after resumed incubations for 3 h samples. The cell lysates were used to infect HEK 293 cells for 48 h and the cells were stained for hexon expression using the protocol as described previously.[12,58,59] The antibody we used for hexon staining is Anti-Adenovirus 1,2,5,6 Antibody, clone 7/48-7a (Cat#: MAB8044, Lot#: 3760555, Sigma, St. Louis, MO). Brown hexon-expressing positive cells were photographed under a microscope, and at least five different fields were counted to quantify the viral replication. Viral burst size, an increase of hexon-expressing cells from 3 to 48 h, was used as an indicator of viral replication.

Adenoviral cytotoxicity assay

MDA-MB-231, MCF-7 cells, or 4T1 cells were seeded into 96-well plates at a density of 1.0×10^3 cells per well. The next day, cells were infected with various doses of adenoviral vectors, ranging from 16 to 1.25×10^6 VPs/cell, and the incubations were continued for 7 days. Cell survival was determined by the sulforhodamine B staining assay as previously described.[12,31,58,60] Untreated control cells were considered to have 100% survival.

sTGFβRIIFc and GM-CSF expression *in vitro*

MDA-MB-231, MCF-7 cells, or 4T1-luc cells were seeded into six-well plates at a density of 2.5×10^5 cells per well. The next day, cells were infected with adenoviral vectors (2.5×10^5 VPs per cell for a total of 5.0×10^5 cells each well) for 24 h. Then, media were changed to serum-free media, incubations continued for another 24 h, and media were collected for ELISA to dectect secreted proteins as previously described.[12,58] The key agents we used for sTGFβRIIFc ELISA are as follows: capture - Human TGF-β RII Antibody (Cat#: AF-241-NA, Lot#: PV1518091, R&D Systems, Minneapolis, MN), Detection - Biotinylated anti-TGF-β RII Antibody (Cat#: BAF241, Lot#: XL0308031, R&D Systems, Minneapolis, MN), and Standard - Recombinant Human TGFβRIIFc Chimera Protein (Cat#: 314-BR, Lot#: OR1211071, R&D Systems, Minneapolis, MN). For GM-CSF ELISA, we used ELISA capture - Human GM-CSF Antibody clone 6804 (Cat#: MAB615, Lot#: ASX2823121, R&D Systems, Minneapolis, MN), Detection - Biotinylated anti-GM-CSF antibody, clone 3209 (Cat#: BAM215, Lot#: ALG1213121, R&D Systems, Minneapolis, MN), and Standard - Recombinant Human GM-CSF Protein (Cat#: 215-GM, Lot#: MAB131311, R&D Systems, Minneapolis, MN).

Animal studies

All animal experimental procedures were approved by the Institutional Animal Care and Use Committee (IACUC) at NorthShore University Health System. Due to adenovirus exposure, mice were housed in a BSL-2 biocontainment room with biohazard-labeled cages and soft bedding. To minimize distress, moistened food was placed on the cage floor or in hanging feeders, and water bottles with extra-long dispensers were used. Animal health was closely monitored using a body condition (BC) score and other clinical indicators. Mice meeting early euthanasia criteria were excluded from the study.

Tumor formation and adenoviral/ICI treatments

To establish the mouse mammary tumor syngeneic mouse model, we injected 2.0×10^6 4T1 (survival analysis) or 4T1-luc (safety and efficacy studies) cells per mouse subcutaneously (day 0) into the dorsal right flank of female BALB/c mice (6–8 weeks old). The subcutaneous tumors were usually apparent after day 6, based on our previous experience for this.[20,23,24] On days 7 and 9 post tumor cell inoculation, saline or adenoviruses were injected into the tumors. For the first set of experiments comparing rAd.sT.GM to rAd.null, rAd.sT, and rAd.GM, 5.0×10^{10} VPs of adenoviruses per mouse for each injection were applied (a total of 1.0×10^{11} VPs for each mouse, five mice per treatment group for day 10 analysis and 12–13 mice per treatment group for day 25 analysis) (Figure S2A). We picked day 10 as our safety analysis endpoint because viral toxicity 24 h after the second injection is most evident from our past studies.[9–12,20,23,24] Also, because the 4T1 tumor is highly aggressive and often grows with ulceration and severe necrosis on the primary injection sites, we had to

start to euthanize some mice after day 20, given severe health concerns and ulceration were present. Thus, day 25 is the most applicable efficacy analysis endpoint based on our extensive experience with this model.[20,23,24] For the second set of experiments investigating combination effects of rAd.sT.GM with ICIs, 2.5×10^{10} VPs of rAd.sT.GM or 100 μL of saline per mouse for each injection were applied to the groups as indicated in the results section (a total of 5.0×10^{10} VPs for each mouse, 11–12 mice per treatment group) (Figure S2B). For the combination therapy experiment, on days 8, 10, 12, and 16, saline or anti-mouse PD-1 antibody Clone RMP1-14 (Cat#: BE0146, Lot#: 810421D1, Bio X Cell, Lebanon, NH) and/or anti-CTLA-4 antibody Clone 9H10 (Cat#: BE0131, Lot#: 755621D1, Bio X Cell, Lebanon, NH)) (0.2 mg per mouse per antibody each time) were administered intraperitoneally. In the single-agent study, to obtain more information of the toxicity profile and access more potential efficacy benefits, 5.0×10^{10} VPs each injection per mouse was used; in the combination study, the regular doses (2.5×10^{10} VPs each injection per mouse) that were previously used for other studies using TERTp promoter-based adenoviruses were applied.[10,11,20,23] No randomization method was used for allocating animals to different experimental groups, since no significant difference of tumor size on day 7 has ever been observed using our optimized tumor cell inoculation protocol.[20,23,24] Several additional mice that were not injected with any tumor cells and viruses served as the normal control. For survival analysis, the same treatment regimen as the combination study was used (12 mice each group), except that 4T1 cells, instead of 4T1-luc cells, were used for tumor inoculation, since we do not need to monitor lung luminescence.

Therapeutic analysis and sample preparation

On day 6 post tumor cell inoculation, tumor dimension was measured (in mm) by using a caliper. Tumor volumes were calculated by the following formula: $(\text{width}^2 \times \text{length})/2$. The tumor volumes were monitored again on indicated days as shown in the results section, together with their body weights. Mice were monitored carefully every day for significant stress/sick signs or severely ulcerated tumors according to our animal protocol. On day 10, blood, spleen, and tumor tissues were collected for safety and efficacy analysis; on day 25, all mice were euthanized, and blood, spleen, lung, and tumor tissues were collected, processed immediately, or frozen for later use according to lab procedures.[23,24] Tumor weights and numbers of lung surface metastatic nodules were also recorded. Parts of lung were fixed, sectioned, and prepared for H&E staining, and they were examined by a Nikon Eclipse TE200 Inverted Microscope and analyzed as described in our previous publications.[23,24] Lung tissue lysates were made from parts of frozen samples by the manufacturer's protocol for Luciferase Assay System (Cat#: E4530, Promega, Madison, WI) and analyzed accordingly for lung metastasis burden. Tumor volume and weight were measured blindly by researchers without knowing which animal group.

Toxicity studies and blood immune marker analysis

Serum levels of lactate dehydrogenase (LDH), aspartate aminotransferase (AST), and alanine transaminase (ALT) were measured using commercially available kits as described in the previous publications.[20,24,58,61] Blood IL-6, TNF-α, IL-1β, sTGFβRIIFc, GM-CSF, and TGFβ-1 expression were measured using the lab ELISA method described previously.[20,24,58,61] Serum samples from the combination treatment experiment were also submitted for additional cytokine analysis using the MSD V-PLEX Mouse Cytokine 19-Plex Kit (PBL Assay Science, Piscataway, NJ) as indicated in the results section.

Quantitative real-time RT-PCR analysis of gene expression

Total RNA was extracted from mouse lung, spleen, and tumor tissues, and cDNA was synthesized using the qScript cDNA SuperMix (catalog#: 101414-102, VWR International, Inc., Radnor, PA) following the manufacturer's instructions. The mRNA expression of various genes in these tissues was measured using the method described in our previous publications.[20,23,24] Details on primer efficiency and specificity were also provided previously.[24] Relative expression (RQ-fold changes) of target genes was calculated using the ΔΔCT method, with normal or buffer samples as calibrators.

Statistical analysis

All statistical analyses were conducted using GraphPad Prism software 9 (version 9.3.1). Group comparisons were performed using one-way ANOVA (Kruskal-Wallis or ordinary) with Dunn's or Bonferroni's multiple comparisons tests, while unpaired t tests (parametric tests and/or non-parametric Mann-Whitney *U* tests) were used for comparing two categorical groups. For outcomes measured repeatedly over time, such as tumor volume growth, two-way ANOVA with Tukey's multiple comparisons tests was utilized. Additionally, F tests were employed to compare variances among and between groups, and those with similar variances were statistically compared and presented. Survival data were statistically analyzed using the Log rank (Mantel-Cox) tests. Significant differences are indicated as follows: $^*p < 0.05$, $^{**}p < 0.01$, $^{***}p < 0.001$, $^{****}p < 0.0001$.

DATA AND CODE AVAILABILITY

All data generated and/or analyzed during this study are included and/or mentioned in the manuscript. The datasets are available from the corresponding author on request.

ACKNOWLEDGMENTS

We greatly appreciate Dr. Prem Seth who started the Cancer Gene Program at NorthShore nearly 20 years ago and founded Amunbio to commercialize rAd.sT.GM as AMUN-003 for new treatments of cancers. We dedicate this publication in his memory. We are very grateful to the Amunbio team that supports us with funding for this study and the Amunbio scientific advisory board that provides valuable comments and suggestions. They are Dr. V.K. Gadi, Ms. Cecilia Zapata-Harms, Mr. Prashanth Chintanapalli, Mr. David Lawrence, and Mr. David Phinney. We are also thankful to our NorthShore (now Endeavor Health) team, for the leadership's support by Dr. Michael Caplan and Dr. Janardan Khandekar, and administration assistance by Dr. Stephen Wachtel and Mr. Robert Stanton.

AUTHOR CONTRIBUTIONS

N.T.T.N. wrote the manuscript with mentorship support from W.X. W.X., S.C.S., and B.F. performed the experiments designed by W.X. Y.Y. and Z.H. previously constructed adenoviruses in this study. B.B. conceptualized this project with Dr. Prem Seth in the past, and also evaluated our final data and manuscript. All authors have directly accessed and verified the data reported in the manuscript.

DECLARATION OF INTERESTS

This study was supported by funds from Amunbio, a biotechnology company developing and commercializing cutting-edge, engineered immunotherapeutic oncolytic viruses for the treatment of cancer and hematological malignancies.

DECLARATION OF GENERATIVE AI AND AI-ASSISTED TECHNOLOGIES IN THE WRITING PROCESS

During the preparation of the manuscript, N.T.T.N. used ChatGPT in order to ensure the clarity, coherence, and correctness of the English writing, and edited the content as needed. W.X. and all authors reviewed the manuscript thoroughly and provided constructive feedback during N.T.T.N.'s writing. All authors take full responsibility for the content of the publication.

SUPPLEMENTAL INFORMATION

Supplemental information can be found online at https://doi.org/10.1016/j.omton.2025.200936.

REFERENCES

1. Bianchini, G., Balko, J.M., Mayer, I.A., Sanders, M.E., and Gianni, L. (2016). Triple-negative breast cancer: challenges and opportunities of a heterogeneous disease. Nat. Rev. Clin. Oncol. *13*, 674–690.
2. Bianchini, G., De Angelis, C., Licata, L., and Gianni, L. (2022). Treatment landscape of triple-negative breast cancer - expanded options, evolving needs. Nat. Rev. Clin. Oncol. *19*, 91–113.
3. Soliman, H., Hogue, D., Han, H., Mooney, B., Costa, R., Lee, M.C., Niell, B., Williams, A., Chau, A., Falcon, S., et al. (2023). Oncolytic T-VEC virotherapy plus neoadjuvant chemotherapy in nonmetastatic triple-negative breast cancer: a phase 2 trial. Nat. Med. *29*, 450–457.
4. Shalhout, S.Z., Miller, D.M., Emerick, K.S., and Kaufman, H.L. (2023). Therapy with oncolytic viruses: progress and challenges. Nat. Rev. Clin. Oncol. *20*, 160–177.
5. Lin, D., Shen, Y., and Liang, T. (2023). Oncolytic virotherapy: basic principles, recent advances and future directions. Signal Transduct. Targeted Ther. *8*, 156.
6. Bommareddy, P.K., Shettigar, M., and Kaufman, H.L. (2018). Integrating oncolytic viruses in combination cancer immunotherapy. Nat. Rev. Immunol. *18*, 498–513.
7. Osipov, I.D., Vasikhovskaia, V.A., Zabelina, D.S., Kutseikin, S.S., Grazhdantseva, A.A., Kochneva, G.V., Davydova, J., Netesov, S.V., and Romanenko, M.V. (2023). Development of Oncolytic Vectors Based on Human Adenovirus Type 6 for Cancer Treatment. Viruses *15*, 182.
8. Alessandrini, F., Menotti, L., Avitabile, E., Appolloni, I., Ceresa, D., Marubbi, D., Campadelli-Fiume, G., and Malatesta, P. (2019). Eradication of glioblastoma by immuno-virotherapy with a retargeted oncolytic HSV in a preclinical model. Oncogene *38*, 4467–4479.
9. Xu, W., Neill, T., Yang, Y., Hu, Z., Cleveland, E., Wu, Y., Hutten, R., Xiao, X., Stock, S.R., Shevrin, D., et al. (2015). The systemic delivery of an oncolytic adenovirus expressing decorin inhibits bone metastasis in a mouse model of human prostate cancer. Gene Ther. *22*, 247–256.
10. Zhao, H., Wang, H., Kong, F., Xu, W., Wang, T., Xiao, F., Wang, L., Huang, D., Seth, P., Yang, Y., and Wang, H. (2019). Oncolytic Adenovirus rAd.DCN Inhibits Breast Tumor Growth and Lung Metastasis in an Immune-Competent Orthotopic Xenograft Model. Hum. Gene Ther. *30*, 197–210.
11. Liu, Z., Yang, Y., Zhang, X., Wang, H., Xu, W., Wang, H., Xiao, F., Bai, Z., Yao, H., Ma, X., et al. (2017). An Oncolytic Adenovirus Encoding Decorin and Granulocyte Macrophage Colony Stimulating Factor Inhibits Tumor Growth in a Colorectal Tumor Model by Targeting Pro-Tumorigenic Signals and via Immune Activation. Hum. Gene Ther. *28*, 667–680.
12. Yang, Y., Xu, W., Neill, T., Hu, Z., Wang, C.H., Xiao, X., Stock, S.R., Guise, T., Yun, C.O., Brendler, C.B., et al. (2015). Systemic Delivery of an Oncolytic Adenovirus Expressing Decorin for the Treatment of Breast Cancer Bone Metastases. Hum. Gene Ther. *26*, 813–825.
13. Romanenko, M., Osipov, I., Netesov, S.V., and Davydova, J. (2021). Adenovirus Type 6: Subtle Structural Distinctions from Adenovirus Type 5 Result in Essential Differences in Properties and Perspectives for Gene Therapy. Pharmaceutics *13*, 1641.
14. Yamamoto, Y., Nagasato, M., Yoshida, T., and Aoki, K. (2017). Recent advances in genetic modification of adenovirus vectors for cancer treatment. Cancer Sci. *108*, 831–837.
15. Feola, S., Capasso, C., Fusciello, M., Martins, B., Tähtinen, S., Medeot, M., Carpi, S., Frascaro, F., Ylosmäki, E., Peltonen, K., et al. (2018). Oncolytic vaccines increase the response to PD-L1 blockade in immunogenic and poorly immunogenic tumors. OncoImmunology *7*, e1457596.
16. Kanaya, N., Kuroda, S., Kakiuchi, Y., Kumon, K., Tsumura, T., Hashimoto, M., Morihiro, T., Kubota, T., Aoyama, K., Kikuchi, S., et al. (2020). Immune Modulation by Telomerase-Specific Oncolytic Adenovirus Synergistically Enhances Antitumor Efficacy with Anti-PD1 Antibody. Mol. Ther. *28*, 794–804.
17. Zhang, H., Xie, W., Zhang, Y., Dong, X., Liu, C., Yi, J., Zhang, S., Wen, C., Zheng, L., and Wang, H. (2022). Oncolytic adenoviruses synergistically enhance anti-PD-L1 and anti-CTLA-4 immunotherapy by modulating the tumour microenvironment in a 4T1 orthotopic mouse model. Cancer Gene Ther. *29*, 456–465.
18. Yi, M., Li, T., Niu, M., Wu, Y., Zhao, Z., and Wu, K. (2022). TGF-beta: A novel predictor and target for anti-PD-1/PD-L1 therapy. Front. Immunol. *13*, 1061394.
19. Batlle, E., and Massagué, J. (2019). Transforming Growth Factor-beta Signaling in Immunity and Cancer. Immunity *50*, 924–940.
20. Xu, W., Yang, Y., Hu, Z., Head, M., Mangold, K.A., Sullivan, M., Wang, E., Saha, P., Gulukota, K., Helseth, D.L., et al. (2020). LyP-1-Modified Oncolytic Adenoviruses Targeting Transforming Growth Factor beta Inhibit Tumor Growth and Metastases and Augment Immune Checkpoint Inhibitor Therapy in Breast Cancer Mouse Models. Hum. Gene Ther. *31*, 863–880.
21. Li, S., Liu, M., Do, M.H., Chou, C., Stamatiades, E.G., Nixon, B.G., Shi, W., Zhang, X., Li, P., Gao, S., et al. (2020). Cancer immunotherapy via targeted TGF-beta signalling blockade in T(H) cells. Nature *587*, 121–125.
22. Ge, R., Rajeev, V., Ray, P., Lattime, E., Rittling, S., Medicherla, S., Protter, A., Murphy, A., Chakravarty, J., Dugar, S., et al. (2006). Inhibition of growth and metastasis of mouse mammary carcinoma by selective inhibitor of transforming growth factor-beta type I receptor kinase *in vivo*. Clin. Cancer Res. *12*, 4315–4330.
23. Yang, Y., Xu, W., Peng, D., Wang, H., Zhang, X., Wang, H., Xiao, F., Zhu, Y., Ji, Y., Gulukota, K., et al. (2019). An Oncolytic Adenovirus Targeting Transforming Growth Factor beta Inhibits Protumorigenic Signals and Produces Immune Activation: A Novel Approach to Enhance Anti-PD-1 and Anti-CTLA-4 Therapy. Hum. Gene Ther. *30*, 1117–1132.
24. Shin, S.C., Vickman, R.E., Filimon, B., Yang, Y., Hu, Z., Mangold, K.A., Prabhakar, B.S., Schreiber, H., and Xu, W. (2024). The safety and efficacy of systemic delivery of a new liver-de-targeted TGFbeta signaling inhibiting adenovirus in an immunocompetent triple negative mouse mammary tumor model. Cancer Gene Ther. *31*, 574–585.
25. Yu, W.L., and Hua, Z.C. (2018). Evaluation of effectiveness of granulocyte-macrophage colony-stimulating factor therapy to cancer patients after chemotherapy: a meta-analysis. Oncotarget *9*, 28226–28239.
26. Lazarus, H.M., Ragsdale, C.E., Gale, R.P., and Lyman, G.H. (2021). Sargramostim (rhu GM-CSF) as Cancer Therapy (Systematic Review) and An Immunomodulator. A Drug Before Its Time? Front. Immunol. *12*, 706186.
27. Salgia, R., Lynch, T., Skarin, A., Lucca, J., Lynch, C., Jung, K., Hodi, F.S., Jaklitsch, M., Mentzer, S., Swanson, S., et al. (2003). Vaccination with irradiated autologous tumor cells engineered to secrete granulocyte-macrophage colony-stimulating factor augments antitumor immunity in some patients with metastatic non-small-cell lung carcinoma. J. Clin. Oncol. *21*, 624–630.
28. Soares, K.C., Rucki, A.A., Kim, V., Foley, K., Solt, S., Wolfgang, C.L., Jaffee, E.M., and Zheng, L. (2015). TGF-beta blockade depletes T regulatory cells from metastatic pancreatic tumors in a vaccine dependent manner. Oncotarget *6*, 43005–43015.
29. Liu, Q., Wu, H., Li, Y., Zhang, R., Kleeff, J., Zhang, X., Cui, M., Liu, J., Li, T., Gao, J., et al. (2020). Combined blockade of TGf-beta1 and GM-CSF improves chemotherapeutic effects for pancreatic cancer by modulating tumor microenvironment. Cancer Immunol. Immunother. *69*, 1477–1492.

30. Yuan, X., Larsson, C., and Xu, D. (2019). Mechanisms underlying the activation of TERT transcription and telomerase activity in human cancer: old actors and new players. Oncogene *38*, 6172–6183.

31. Dai, S., Lv, Y., Xu, W., Yang, Y., Liu, C., Dong, X., Zhang, H., Prabhakar, B.S., Maker, A.V., Seth, P., and Wang, H. (2020). Oncolytic adenovirus encoding LIGHT (TNFSF14) inhibits tumor growth via activating anti-tumor immune responses in 4T1 mouse mammary tumor model in immune competent syngeneic mice. Cancer Gene Ther. *27*, 923–933.

32. Yao, X., Yoshioka, Y., Eto, Y., Morishige, T., Okada, Y., Mizuguchi, H., Mukai, Y., Okada, N., and Nakagawa, S. (2007). TERT promoter-driven adenovirus vector for cancer gene therapy via systemic injection. Biochem. Biophys. Res. Commun. *362*, 419–424.

33. Wirth, T., Zender, L., Schulte, B., Mundt, B., Plentz, R., Rudolph, K.L., Manns, M., Kubicka, S., and Kühnel, F. (2003). A telomerase-dependent conditionally replicating adenovirus for selective treatment of cancer. Cancer Res. *63*, 3181–3188.

34. Luo, Y., Lin, C., Zou, Y., Ju, F., Ren, W., Lin, Y., Wang, Y., Huang, X., Liu, H., Yu, Z., et al. (2020). Tumor-targeting oncolytic virus elicits potent immunotherapeutic vaccine responses to tumor antigens. OncoImmunology *9*, 1726168.

35. Kawashima, T., Kagawa, S., Kobayashi, N., Shirakiya, Y., Umeoka, T., Teraishi, F., Taki, M., Kyo, S., Tanaka, N., and Fujiwara, T. (2004). Telomerase-specific replication-selective virotherapy for human cancer. Clin. Cancer Res. *10*, 285–292.

36. Renaud-Gabardos, E., Hantelys, F., Morfoisse, F., Chaufour, X., Garmy-Susini, B., and Prats, A.C. (2015). Internal ribosome entry site-based vectors for combined gene therapy. World J. Exp. Med. *5*, 11–20.

37. Simmons, A.D., Li, B., Gonzalez-Edick, M., Lin, C., Moskalenko, M., Du, T., Creson, J., VanRoey, M.J., and Jooss, K. (2007). GM-CSF-secreting cancer immunotherapies: preclinical analysis of the mechanism of action. Cancer Immunol. Immunother. *56*, 1653–1665.

38. Burgess, A.W., and Metcalf, D. (1977). Serum half-life and organ distribution of radiolabeled colony stimulating factor in mice. Exp. Hematol. *5*, 456–464.

39. Hovgaard, D., Mortensen, B.T., Schifter, S., and Nissen, N.I. (1993). Comparative pharmacokinetics of single-dose administration of mammalian and bacterially-derived recombinant human granulocyte-macrophage colony-stimulating factor. Eur. J. Haematol. *50*, 32–36.

40. Thakur, B., Saha, L., and Bhatia, A. (2022). Relative refractoriness of breast cancer cells to tumour necrosis factor-alpha induced necroptosis. Clin. Exp. Pharmacol. Physiol. *49*, 1294–1306.

41. Singh, K., Poteryakhina, A., Zheltukhin, A., Bhatelia, K., Prajapati, P., Sripada, L., Tomar, D., Singh, R., Singh, A.K., Chumakov, P.M., and Singh, R. (2015). NLRX1 acts as tumor suppressor by regulating TNF-alpha induced apoptosis and metabolism in cancer cells. Biochim. Biophys. Acta *1853*, 1073–1086.

42. Kee, J.Y., Ito, A., Hojo, S., Hashimoto, I., Igarashi, Y., Tsuneyama, K., Tsukada, K., Irimura, T., Shibahara, N., Takasaki, I., et al. (2014). CXCL16 suppresses liver metastasis of colorectal cancer by promoting TNF-alpha-induced apoptosis by tumor-associated macrophages. BMC Cancer *14*, 949.

43. Schioppa, T., Moore, R., Thompson, R.G., Rosser, E.C., Kulbe, H., Nedospasov, S., Mauri, C., Coussens, L.M., and Balkwill, F.R. (2011). B regulatory cells and the tumor-promoting actions of TNF-alpha during squamous carcinogenesis. Proc. Natl. Acad. Sci. USA *108*, 10662–10667.

44. Chopra, M., Riedel, S.S., Biehl, M., Krieger, S., von Krosigk, V., Bäuerlein, C.A., Brede, C., Jordan Garrote, A.L., Kraus, S., Schäfer, V., et al. (2013). Tumor necrosis factor receptor 2-dependent homeostasis of regulatory T cells as a player in TNF-induced experimental metastasis. Carcinogenesis *34*, 1296–1303.

45. Hodi, F.S. (2024). A Phase II/III Trial of Nivolumab, Ipilimumab, and GM-CSF in Patients With Advanced Melanoma. https://clinicaltrials.gov/study/NCT02339571?term=NCT02339571&rank=1.

46. Jakub, J. (2020). Neoadjuvant Granulocyte-Macrophage Colony-Stimulating Factor (GM-CSF) in Cutaneous Stage L-lll Melanoma. https://clinicaltrials.gov/study/NCT02451488?term=NCT02451488&rank=1.

47. Olivares, J., Kumar, P., Yu, Y., Maples, P.B., Senzer, N., Bedell, C., Barve, M., Tong, A., Pappen, B.O., Kuhn, J., et al. (2011). Phase I trial of TGF-beta 2 antisense GM-CSF gene-modified autologous tumor cell (TAG) vaccine. Clin. Cancer Res. *17*, 183–192.

48. Tauriello, D.V.F., Palomo-Ponce, S., Stork, D., Berenguer-Llergo, A., Badia-Ramentol, J., Iglesias, M., Sevillano, M., Ibiza, S., Cañellas, A., Hernando-Momblona, X., et al. (2018). TGFbeta drives immune evasion in genetically reconstituted colon cancer metastasis. Nature *554*, 538–543.

49. Fix, S.M., Forget, M.A., Sakellariou-Thompson, D., Wang, Y., Griffiths, T.M., Lee, M., Haymaker, C.L., Dominguez, A.L., Basar, R., Reyes, C., et al. (2022). CRISPR-mediated TGFBR2 knockout renders human ovarian cancer tumor-infiltrating lymphocytes resistant to TGF-beta signaling. J. Immunother. Cancer *10*, e003750.

50. Gong, L., Ji, L., Xu, D., Wang, J., and Zou, J. (2021). TGF-beta links glycolysis and immunosuppression in glioblastoma. Histol. Histopathol. *36*, 1111–1124.

51. Monge, C., Xie, C., Myojin, Y., Coffman, K., Hrones, D.M., Wang, S., Hernandez, J.M., Wood, B.J., Levy, E.B., Juburi, I., et al. (2023). Phase I/II study of PexaVec in combination with immune checkpoint inhibition in refractory metastatic colorectal cancer. J. Immunother. Cancer *11*, e005640.

52. Zeng, M., Zhang, W., Li, Y., and Yu, L. (2024). Harnessing adenovirus in cancer immunotherapy: evoking cellular immunity and targeting delivery in cell-specific manner. Biomark. Res. *12*, 36.

53. Leikas, A.J., Yla-Herttuala, S., and Hartikainen, J.E.K. (2023). Adenoviral Gene Therapy Vectors in Clinical Use-Basic Aspects with a Special Reference to Replication-Competent Adenovirus Formation and Its Impact on Clinical Safety. Int. J. Mol. Sci. *24*, 16519.

54. Horn, J. (2024). Study of Cretostimogene Given in Patients with Non-muscle Invasive Bladder Cancer (Unresponsive to Bacillus-Calmette-Guerin (BOND-003)). https://clinicaltrials.gov/study/NCT04452591?term=NCT04452591&rank=1.

55. Leslie, M. (2023). Tumor-killing viruses score rare success in late-stage trial. Science *382*, 1101–1102.

56. Seth, P., Wang, Z.G., Pister, A., Zafar, M.B., Kim, S., Guise, T., and Wakefield, L. (2006). Development of oncolytic adenovirus armed with a fusion of soluble transforming growth factor-beta receptor II and human immunoglobulin Fc for breast cancer therapy. Hum. Gene Ther. *17*, 1152–1160.

57. Hu, Z., Robbins, J.S., Pister, A., Zafar, M.B., Zhang, Z.W., Gupta, J., Lee, K.J., Newman, K., Yun, C.O., Guise, T., and Seth, P. (2010). A modified hTERT promoter-directed oncolytic adenovirus replication with concurrent inhibition of TGFbeta signaling for breast cancer therapy. Cancer Gene Ther. *17*, 235–243.

58. Xu, W., Zhang, Z., Yang, Y., Hu, Z., Wang, C.H., Morgan, M., Wu, Y., Hutten, R., Xiao, X., Stock, S., et al. (2014). Ad5/48 hexon oncolytic virus expressing sTGFbetaRIIFc produces reduced hepatic and systemic toxicities and inhibits prostate cancer bone metastases. Mol. Ther. *22*, 1504–1517.

59. Hu, Z., Gupta, J., Zhang, Z., Gerseny, H., Berg, A., Chen, Y.J., Zhang, Z., Du, H., Brendler, C.B., Xiao, X., et al. (2012). Systemic delivery of oncolytic adenoviruses targeting transforming growth factor-beta inhibits established bone metastasis in a prostate cancer mouse model. Hum. Gene Ther. *23*, 871–882.

60. Wang, Z.G., Zhao, W., Ramachandra, M., and Seth, P. (2006). An oncolytic adenovirus expressing soluble transforming growth factor-beta type II receptor for targeting breast cancer: *in vitro* evaluation. Mol. Cancer Therapeut. *5*, 367–373.

61. Zhang, Z., Krimmel, J., Zhang, Z., Hu, Z., and Seth, P. (2011). Systemic delivery of a novel liver-detargeted oncolytic adenovirus causes reduced liver toxicity but maintains the antitumor response in a breast cancer bone metastasis model. Hum. Gene Ther. *22*, 1137–1142.

OMTON, Volume *33*

Supplemental information

Oncolytic adenovirus inhibits TNBC tumor growth/metastasis in mice by targeting TGF-β and overexpressing GM-CSF

Nguyễn Thị Thanh Nhàn, Soon Cheon Shin, Beniamin Filimon, Yuefeng Yang, Zebin Hu, Bruce Brockstein, and Weidong Xu

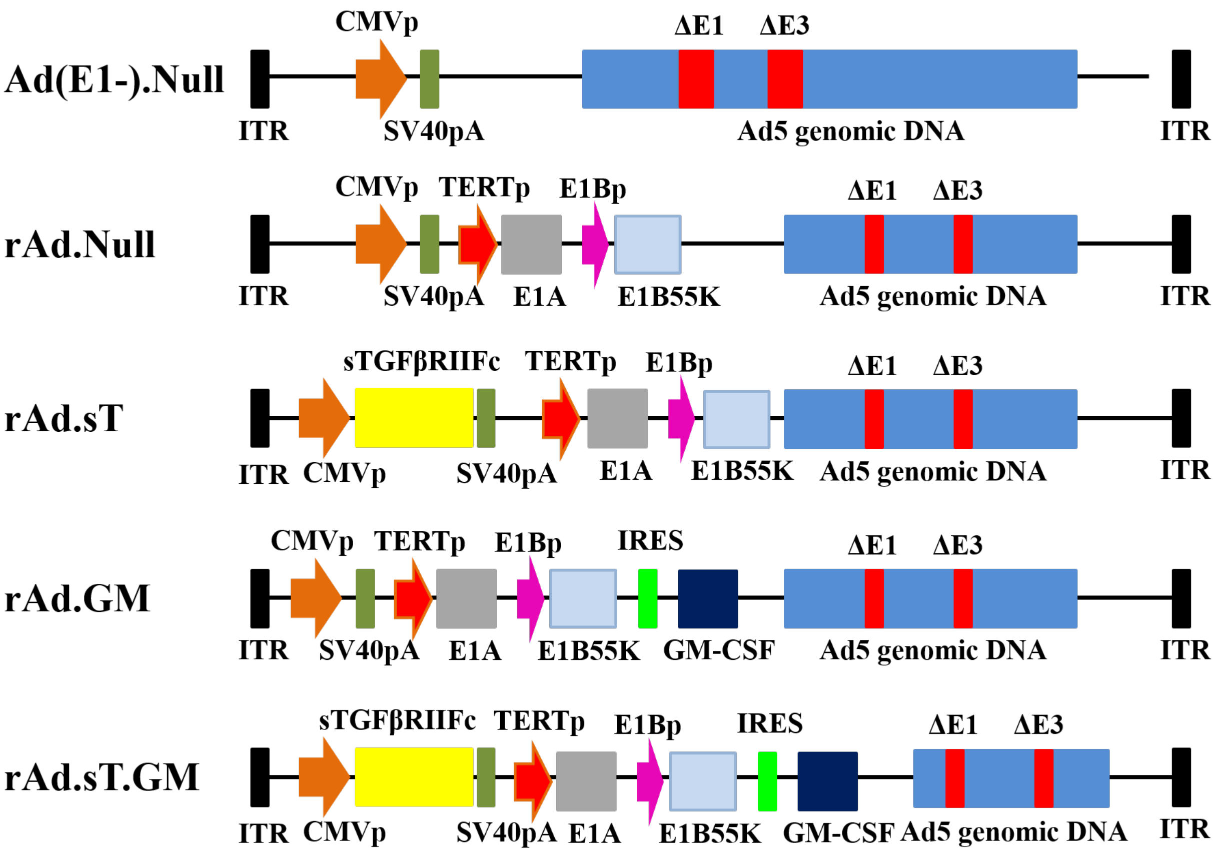

Figure S1: A schematic diagram of key genes in Ad(E-).null, rAd.null, rAd.sT, rAd.GM, and rAd.sT.GM

ITR: Inverted Terminal Repeat containing the cis-acting DNA sequences that is important in the initiation of viral DNA replication and binding to the cellular transcription factors SP1 and ATF;

CMVp: human cytomegalovirus (CMV) promoter for target gene expression;

SV40 pA: a SV40 polyadenylation site increasing mRNA stability;

TERTp: Telomerase reverse transcriptase (TERT) promoter allowing target gene expression in cells with high telomerase activity;

E1A: Adenovirus early region 1A (E1A) producing E1A protein to induce DNA replication;

E1Bp: Adenovirus early region 1B (E1B) promoter allowing E1B55K expression;

E1B55K: The gene for 55-kDa E1B protein that supports productive adenoviral infection and

replication;

IRES: Internal ribosome entry site allowing the expression of additional target genes. It is an RNA element that allows for translation initiation in the absence of a 5' cap for stable expression in cells by viral vectors;

ΔE1: The deletion of the early region 1 (E1) gene which encodes E1A and E1B proteins required for adenoviral replication and a productive lytic cycle.

ΔE3: The deletion of the early region 3 (E3) gene that expresses E3 proteins that manipulate the host immune response for immune evasion.

The expression of the therapeutic (target) genes: sTGFβRIIFc and GM-CSF is mediated either by the CMV promoter or IRES, respectively.

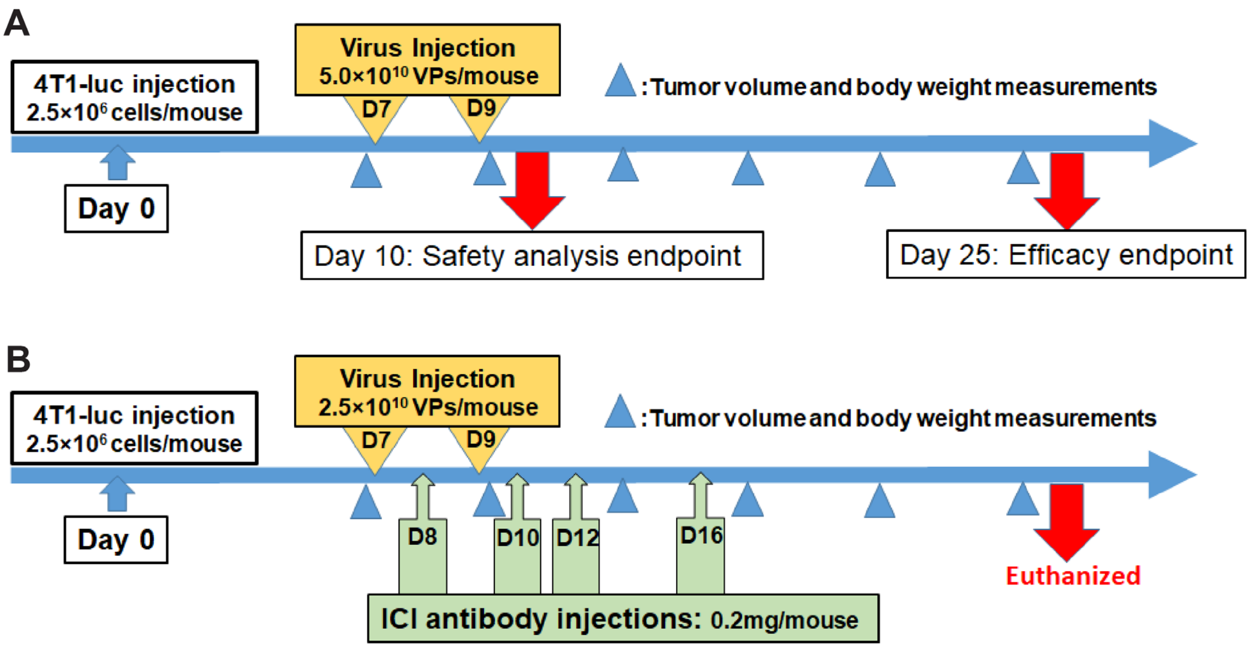

Figure S2: Treatment regimens. (A) The single agent study: Female BALB/c mice (6-8 weeks old) were subcutaneously injected with 2.0×10^{6} 4T1-luc cells per mouse into the dorsal right flank on day 0. Tumors became visible by day 6. On days 7 and 9 post-tumor cell inoculation, two doses of saline or adenoviruses (5.0×10^{10} VPs per mouse each injection) were injected directly into the tumors. 5 mice per treatment group were sacrificed on day 10 for safety analysis, and 12-13 mice per group were monitored with animal health conditions and periodic tumor caliper for tumor sizes, and sacrificed on Day 25 for the efficacy study. At all terminal points, blood, tumor and tissue samples were collected for analysis; (B) The combination study: Using the same animal model with a reduced dose of rAd.sT.GM (2.5×10^{10} VPs per mouse each injection), and with the combination of anti-PD-1 antibody, anti-CTLA-4 antibodies, or both (0.2 mg of antibody per mouse each injection, IP, on days 8, 10, 12, and 16), the efficacy of the combination treatment was evaluated. 11-12 mice per treatment group and all of them were carefully monitored for their health conditions and tumor volumes. They were sacrificed on Day 25 with specimens collected as described above.

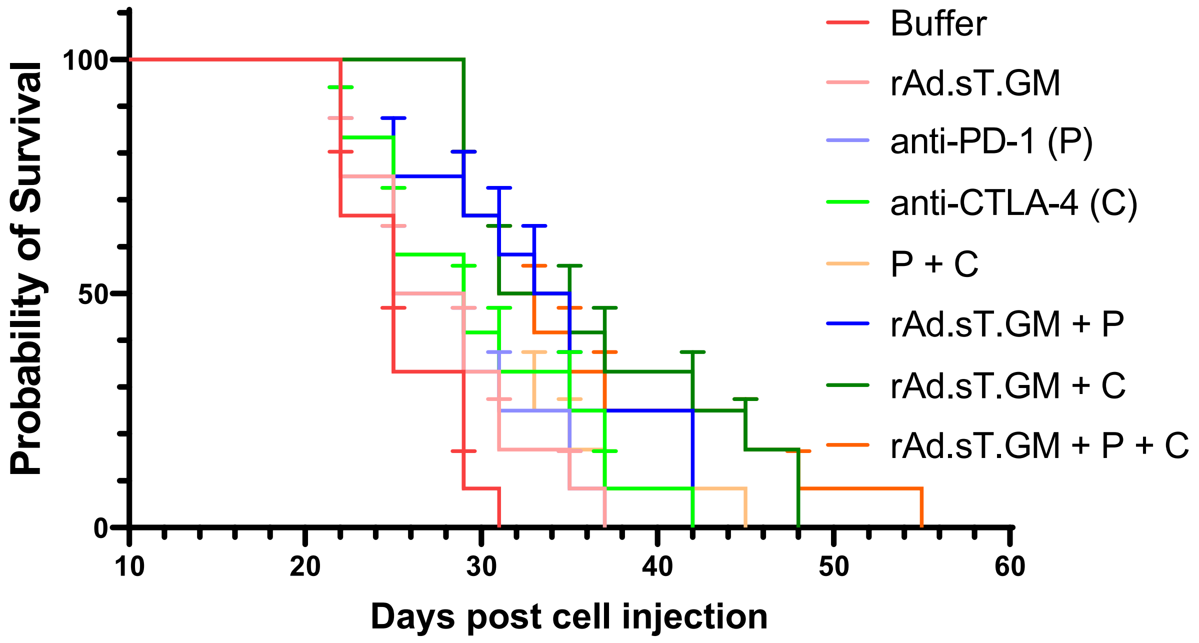

Figure S3: Kaplan-Meier curves displaying the overall survival of subjects across different treatment groups (n=12). Survival data were statistically analyzed using the Log-rank (Mantel-Cox) tests, with significant differences between the Buffer control and treatment groups indicated below:

Buffer vs anti-CTLA-4: $p = 0.0369$ (*)

Buffer vs anti-PD-1 + anti-CTLA-4: $p = 0.0369$ (*)

Buffer vs rAd.sT.GM + anti-PD-1: $p = 0.0019$ (**)

Buffer vs rAd.sT.GM + anti-CTLA-4: $p = 0.0001$ (***)

Buffer vs rAd.sT.GM + anti-PD-1 + anti-CTLA-4: $p = 0.0001$ (***)

Significance is indicated as *= $p < 0.05$, **= $p < 0.01$, ***= $p < 0.001$.

Table S1: Comparsions of treatment efficacy of rAd.sT.GM to other treatments and the buffer group in the single agent study. Significance is indicated as *= $p < 0.05$, **= $p < 0.01$, ***= $p < 0.001$, ****= $p < 0.0001$.

Measurements	Statistical analysis methods	rAd.sT.GM vs.				Notes
		Buffer	rAd.null	rAd.sT	rAd.GM	
Tumor volume progression	Two-way ANOVA	****	ns	***	ns	Day 21
		****	ns	****	*	Day 25
Tumor weight	One-way ANOVA	****	ns	ns	ns	Day 25
	t-test	****	ns	ns	ns	
Lung metastatic nodules	One-way ANOVA	ns	*	ns	ns	Day 25
	t-test	*	***	ns	ns	
Lung luminescense	One-way ANOVA	**	ns	ns	ns	
	t-test	***	*	ns	ns	

Table S2: Comparsions of treatment efficacy of rAd.sT.GM-ICIs combination to other treatments and the buffer group in the combination study on day 25. Significance is indicated as *= $p < 0.05$, **= $p < 0.01$, ***= $p < 0.001$, ****= $p < 0.0001$.

Measurement	Statistical analysis methods	rAd.sT.GM + P + C vs.						
		Buffer	rAd.sT.GM	anti-PD-1 (P)	anti-CTLA-4 (C)	P + C	rAd.sT.GM + P	rAd.sT.GM + C
Tumor volume ratio	One-way ANOVA	****	***	***	ns	ns	***	ns
	t-test	****	***	****	**	*	****	ns
Tumor weight	One-way ANOVA	ns	ns	*	ns	ns	ns	ns
	t-test	*	**	**	ns	ns	ns	ns
Lung metastatic nodules	One-way ANOVA	****	ns	***	ns	ns	ns	ns
	t-test	****	*	****	***	***	**	ns
Lung metastatic area	One-way ANOVA	****	*	**	ns	ns	ns	ns
	t-test	****	**	**	*	ns	ns	ns
Lung luminescense	One-way ANOVA	**	ns	ns	ns	ns	ns	ns
	t-test	*	ns	*	ns	ns	ns	ns
